# Standard assessments of climate forecast skill can be misleading

**DOI:** 10.1038/s41467-021-23771-z

**Published:** 2021-07-16

**Authors:** James S. Risbey, Dougal T. Squire, Amanda S. Black, Timothy DelSole, Chiara Lepore, Richard J. Matear, Didier P. Monselesan, Thomas S. Moore, Doug Richardson, Andrew Schepen, Michael K. Tippett, Carly R. Tozer

**Affiliations:** 1 CSIRO Oceans & Atmosphere, Hobart, TAS Australia; 2 Department of Atmospheric, Oceanic, and Earth Sciences, George Mason University, Fairfax, VA USA; 3 Lamont-Doherty Earth Observatory, Columbia University, Palisades, NY USA; 4 CSIRO Land & Water, Brisbane, QLD Australia; 5 Department of Applied Physics and Applied Mathematics, Columbia University, New York, NY USA

**Keywords:** Climate sciences, Atmospheric dynamics, Projection and prediction

## Abstract

Assessments of climate forecast skill depend on choices made by the assessor. In this perspective, we use forecasts of the El Niño-Southern-Oscillation to outline the impact of bias-correction on skill. Many assessments of skill from hindcasts (past forecasts) are probably overestimates of attainable forecast skill because the hindcasts are informed by observations over the period assessed that would not be available to real forecasts. Differences between hindcast and forecast skill result from changes in model biases from the period used to form forecast anomalies to the period over which the forecast is made. The relative skill rankings of models can change between hindcast and forecast systems because different models have different changes in bias across periods.

## Abstract (web summary)

Many different methods have been developed to forecast climate phenomena like the El Nino-Southern Oscillation (ENSO) which makes a fair comparison of their capabilities crucial. In this perspective, the authors discuss how choices in the evaluation method can lead to an overestimated perceived skill of ENSO forecasts.

## Introduction

Climate forecasts attempt to track the evolution of the more slowly evolving parts of the climate system. They can extend over any time scale longer than weather forecasts^1^, but have been pioneered and applied so far mostly on seasonal timescales. Seasonal climate forecasts aim to predict those processes of climate variability that evolve and carry memory on the seasonal time scale, and which transmit their expression into weather systems and other features of interest^2^. These processes are often related to so called modes of variability, of which the El Niño Southern Oscillation (ENSO)^3–5^ in the tropical Pacific is one of the most important ones for seasonal to interannual climate forecasts. This is mainly because it evolves on seasonal and longer timescales, and it provides discernable influences on weather systems across large swathes of the Pacific and Indian Ocean basins and into the Antarctic region^6^, so called teleconnections^7^.

Here, we discuss the influence a number of methodological decisions can have on the assessment of predictive skill of ENSO forecasts. While we focus on evaluating ENSO forecast skill here, the issues arising in the interaction of forecast processing and skill apply more generally to all climate forecasts.

## History of ENSO forecast skill

Forecasts of ENSO have been issued since the late 1980s^8^, with more routine forecasts emerging in the 1990’s to include those made through coupled general circulation models (CGCMs)^9^. The forecasts from CGCMs sit alongside those from simpler physical models or empirical statistical models that use past climate data to form relationships to predict future outcomes. There is a general expectation in the community that forecast skill from the CGCMs has, or at least will eventually, outstrip that from statistical models^10,11^ (see Supplementary Note 1 for an assessment of how long ENSO forecasts have skill). ENSO is a dynamical process, whereby each event is associated with a form of onset (the event begins), duration, and decay (the event ends). ENSO onset involves a range of interacting nonlinear processes^12,13^ and would seem to be better suited to prediction by dynamical (CGCM) than statistical models^14^. Despite this, it is still unclear whether and when CGCMs are better than statistical models and whether either model type has meaningful skill in forecasting the onset of ENSO.

These questions have been answered differently by different studies. For example, Barnston et al.^10^ concludes that ENSO skill in CGCMs now exceeds that in the statistical models used to forecast ENSO. On the other hand, DelSole and Tippett^15^ finds that a set of CGCMs^16^ are no more skillful than a simple regression model in forecasting ENSO. On the question of forecasting onset, L’Heureux et al.^17^ propose the 2015/16 El Niño as an example of successful ENSO prediction, while Glantz^18^ reviews past El Niño forecasts and concludes that there is little skill in forecasting onset.

There are a range of reasons why there is no real definitive view on ENSO forecast skill in dynamical models. A major difficulty with all skill assessments of ENSO is that there are only a handful of major ENSO events of each type (El Niño, La Niña) in the period since the 1980s and 1990s when ENSO hindcasting and forecasting commenced^13^. The small number of events limits the power of any statistical tests of skill^10,14,19^. The assessment of ENSO forecast skill will depend on the period (typically a decade or two) chosen to test and the number and type of ENSO events in the period^10^. The predictability of ENSO may vary from period to period in the sense that the predictability of chaotic systems varies due to flow dependency^20^. The predictability will also appear to vary for different periods simply because they will contain different very small samples of ENSO behaviour^21^. Another factor that can influence the forecast skill assessment is the inclusion of artificial skill in the evaluation procedure.

## Artificial skill

The apparent forecast skill of ENSO varies depending on whether skill is assessed in hindcasts (forecasts over periods in the past) or forecasts (predictions over yet-to-be observed future periods). Most ENSO skill assessments have been based on hindcasts. Hindcast datasets can contain many more forecasts than actual forecast archives, and so offer a chance for more robust statistics in the assessment of skill. On the other hand, hindcasts present more idealised conditions for forecasts than for actual forecasts because they allow for more complete ingestion of data for initial conditions, tuning and calibration of the model over events that will be included in model tests, and opportunities to provide more systematic post-processing of the forecasts. These differences imply that hindcast skill can be a poor estimate of true forecast skill^21^. Hindcast skill can be an overestimate of real forecast skill if the hindcasts contain artificial skill^22^, or an underestimate if there is variability in skill sampled from different periods. The term ‘artificial skill’ here is understood to refer to skill in hindcasts that would not be attainable in a real forecast system due to some aspect of the idealised nature of the hindcasts; e.g., using data that would not be available in a comparable forecast situation.

The production of artificial skill is important as it can mislead the users of climate forecasts about the true skill and utility of the forecasts for their operations. All climate models have some bias relative to the real world (see Box 1). The post-processing of ENSO hindcasts usually includes a step (or steps) to correct for this bias and to translate the actual forecast values of sea surface temperature into anomalies. Without bias-correction, the model forecast climate may be too far from the observed climate to be readily interpretable. The process of bias-correction is one place where artificial skill can enter into the assessment of hindcast skill. In most ENSO hindcast skill assessments, the bias-correction is carried out in a single step in forming the forecast anomalies. Our focus here is on the methods typically used to generate bias-corrected forecast anomalies, their potential role in generating artificial skill, and how this relates to the diversity of views of ENSO skill. We show this using a simple set of bias-correction methods that are representative of the main methods applied to hindcast data in the literature on ENSO skill.

Our discussion of bias-correction methods (see Box 2) divides them into two broad categories, denoted ‘fair’ and ‘unfair’. These terms are used strictly in regard to what is ‘fair’ in skill comparisons with real-time climate forecasts. Any bias-corrected hindcast that uses observed data that would not be available to a real-time forecast (because the observation occurs after the forecast commences) is classified as ‘unfair’. Such a forecast has an unfair advantage by knowing some aspect of the future that is not knowable to real forecasts. We make this broad distinction between bias-correction methods because it is directly relevant to whether artificial skill enters the forecasts.

Box 1 Model bias Since climate models are not perfect representations of the real world, the climatology of any forecast model (the mean climate formed from a set of the forecasts made) will never reproduce the observed climatology exactly. The time averaged difference between the model and observed climatology is called model bias. For climate forecasts, the model is initialised to start the forecast close to the observations as shown in Fig. Box 1 panel **a** for a sequence of model starts. The model climatology at 0^*^-months lead is therefore close to the observed climatology, as is evident in Fig. Box 1 panel **b**, which shows the near-identical model and observed climatologal distributions for each calendar month of the year. At longer lead times the model forecast will revert towards the model’s preferred climatology. This is evident at lead 11 in Fig. Box 1 panel **c**, by which time the model no longer tracks observations so closely. The climatology of the model at lead 11 now shows clear differences from the observed in Fig. Box 1 panel **d**. The model means for each month have shifted from the observed means, and the shape of the model distribution is different from the observed. The model’s mean bias is typically removed from forecast fields by subtracting the difference between model mean and observed mean climatologies (blue shaded area in Fig. Box 1 panels **b** and **d**) for each month for each model lead time. This correction does not address any differences between the shapes of the model and observed distributions.

Fig. Box 1: Model bias schematic. Panels **a** and **c** show Niño3.4 temperature for observations and the aer04 model ensemble mean at lead 0^*^ (the start of the forecast) and after 11 months, for a sequence of forecasts issued a month apart. The observations values are simply the time series of Niño3.4 temperature and are identical at lead 0* and lead 11. The model values at lead 11 are the values predicted by forecasts initialised 11 months prior to the time shown. The panels **b** and **d** show the distribution of lead 0*   and lead 11 values for the model and observations for each calendar month. These are again identical for observations, but change for the model. The dots on these plots show the mean of the distributions.

Box 2 Bias correction methods When evaluating climate forecasts, the target forecast variable is often converted to anomalies (about some specified mean climatology) for comparison to observations. This process may remove mean model bias over the chosen climatological period. The choices made in forming this climatology can have an impact on the assessment of skill. For assessment of hindcast data, it is good practice to define a period of time to be used as a reference climatology for the bias-correction (a training period), and a separate period to be used for testing the hindcasts (testing period)^43^. We have divided the hindcast archive period into two such periods (1982–1998 & 1999–2015) as shown in schematic form in Fig. Box 2. In practice, model groups make a range of different choices of reference climatology. We summarise the main approaches as follows.

***biased*** develops a climatology in the training period from observations (purple box in Fig. 2)^44–46^. For the *biased* method, observed values of Niño3.4 are averaged as a function of calendar month and used as a climatology to subtract from all matched calendar months in the forecast data. No variation in the climatology is made for lead time here, since there is no lead-time dependence in the observed climatology. By not subtracting the model’s own climatology, any offset or bias in where the model climatology sits relative to observations is retained in the anomalies.

***unfair*** uses a baseline climatology for the forecasts from the set of all forecasts (as a function of calendar month and lead time) in the test period (pink box). Though this method is widely used for deriving forecast anomalies^21,29,30,39,41,46–56^, it is a form of ‘cheating’ in skill evaluation^45^, as it uses observed data in the test period for which the model will be evaluated to adjust the forecast anomalies in that period. The reference climatology in the test period is made up of forecasts initialised in this period, which require observations to initialise. These observations would not be available to actual forecasts.

***unfair-cv*** is a variant of the *unfair* method that applies a form of ‘cross-validation’, whereby a period of time centred around the current forecast time is removed, and then the current anomaly is computed with respect to the climatology of the remaining data^57^ (red boxes). This method is important in the literature as it is often posed as a form of best practice^21,27,33,45^. This method is still in the *unfair* category as it uses information from the future period after the forecast is made.

***fair*** derives the model climatology from the appropriate training period (green box). A ‘fair’ forecast can not use any observations of the climate system that apply to the period after the forecast commences, since such observations are never available for real-time forecasting. Some hindcasts have been assessed with the *fair* method^15,58^, and of course all assessments of real-time forecasts^9,10,59^ are fair. Variants of *fair* include *fair*-*sliding*, which uses a sliding window behind the forecast as a reference period (blue box), and *fair-all* with a growing window that includes all forecasts initiated prior to the current forecast (orange box).

Fig. Box 2: Schematic bias correction plot. The schematic shows a time series of the observed variable at the top and hindcast/forecast time series at different lead times (to 11 months) on the bottom. The time series are divided in half to give a training period (blue shade) and a testing period (tan shade). The training period is typically reserved for computing the model climatology of the forecasts, and the testing period is used to test the bias-corrected forecasts. The coloured lines depict the periods used in forming the reference climatologies for the different bias correction methods described above. Solid lines indicated fixed periods. Dashed lines indicate periods that move as the forecast start date moves.

## Reference forecasts

Forecasts are typically compared with some form of simple reference forecast to set a baseline for skill. We use here a simple linear regression model of ENSO described by DelSole and Tippett^15^ and (in the supplementary information) an ordered logistic regression model^23^ as reference forecasts. The goal here is not to compare CGCMs to sophisticated statistical models or operational statistical models, as in Barnston et al.^10^. Rather, we use simple regression models to provide a clear baseline that has no explicit dynamics or physics, and only very basic fitting to the observed data. We employ two types of regression model because they can be related to different ways of assessing skill. The linear regression model minimises the distance of the data from the fit to the data and thus may have an advantage for any skill score that is based on distance of the forecast from the observations. On the other hand, the ordered logistic regression provides a minimisation based on categories in the data, such as El Niño, neutral, and La Niña conditions, and may perform better in categorical assessments. By engaging both a linear and categorical regression here we can try to account for the advantages of each type of model depending on whether a distance-based or category-based skill score is used. The logistic regression model is only used here for categorical forecasts.

**Fig. 1:**
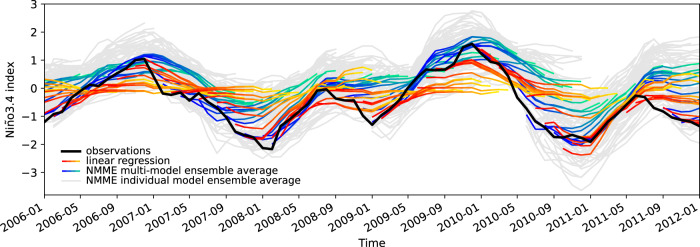
El Niño Southern Oscillation (ENSO) time series and forecasts. The black line shows the observed value of Niño3.4. The blue/green lines are 12-month forecasts of Niño3.4 for the North American Multimodel Ensemble (NMME) and Copernicus Climate Change Service (C3S) multimodel ensemble mean. The shading for these forecast lines runs from dark blue for the first month of the forecast through green for long lead times. The red/yellow lines are 12-month forecasts of Niño3.4 for the linear regression model, where the first month is shaded red and longer leads are yellow. The grey lines are ensemble mean forecasts from individual NMME and C3S models. The *fair* method is used to calculate anomalies.

Regression forecasts (of each type) are generated over the period from 1982 matching the CGCM forecasts. Most forecasts are run a year from initiation. The resolution of the forecasts is monthly, allowing for lead times out to a year. The forecasts are made for the value of Niño3.4, which is a typical index of ENSO. The CGCM forecasts are represented by the ensemble mean of 10-member ensembles for each CGCM. An example of the resulting forecasts is shown in Fig. 1. The peak of Niño3.4 values in the middle of the figure represents the 2010 El Niño event. The CGCM ensemble mean forecasts initialised before the event tend to miss the onset of the event as depicted by the forecasts that continue with low amplitude despite the rising amplitude of the observed event. The tendency to miss the onset of the event is even more apparent for the linear regression model.

## Dynamical models versus regression models

We compare the skill of the set of dynamical model hindcasts/forecasts of Niño3.4 with the skill of the simple regression models. We use a test period of 1999–2016 to assess the model skill. We further divide our skill assessment into cases where the bias-correction of the forecasts uses a training period of 1982–1998 (fair methods), and where it uses forecasts (and observations) from the testing period after the commencement of the forecast (unfair methods). These methods are described in detail in Box 2. The relative skill of the forecast systems is assessed with the random walk skill scores described in the methods section.

**Fig. 2:**
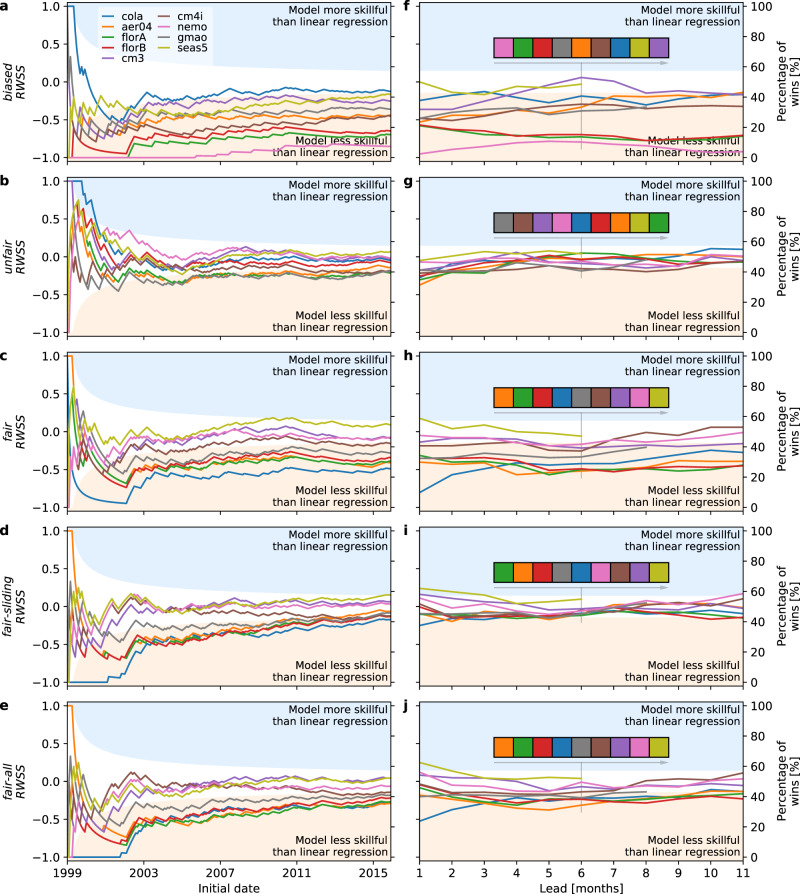
Skill relative to linear regression. Dynamical model Niño3.4 random walk skill score (*RWSS*) at lead 3 months relative to linear regression model using the methods: *biased*, *unfair*, *fair*, *fair-sliding*, and *fair-all* in panels (**a**–**e**). Forecasts are for the period 1999 to 2016. The white shaded area is an envelope encompassing the *RWSS* that would be obtained 95% of the time for coin toss trials. The blue shaded area indicates regions where a given dynamical model would be closer to observations than the regression model more often than expected due to chance. The tan region is the converse where the regression model is more often closer to observations than the dynamical model than expected by chance. The *RWSS* can vary between −1 (model always less skillful than regression), 0 (model and regression equally skillful), to 1 (model always more skillful than regression). This score can also be expressed as the percentage of wins for the dynamical model (right vertical axis). The panels (**f**–**j**) show the final value of the random walk skill score, *RWSS*_*n*_, for lead times from 1–11 months. The coloured boxes in panels (**f**–**j**) are ordered to show the model rankings relative to linear regression at lead 6, going from least skillful on the left to most skillful on the right. The colour code for the models is given in the legend in panel **a**. For skill relative to logistic regression, see Supplementary Note 3.

The results for the random walk comparison at lead time 3 months (a typical seasonal forecast lead) are shown in Fig. 2 panels a–e for each of the bias-correction methods. The results are all relative to the linear regression model. The interpretation of the scores is as follows. When the random walk skill score (*RWSS*) in Fig. 2 is steady and horizontal (zero gradient), the forecast comparisons have stabilised such that the relative proportion of wins for each system is not changing as additional forecasts are included in the comparison. The proportion of wins for the dynamical model forecast can be read from the right vertical axis label. The slope of the *RWSS* line is indicative of any changes in the proportion by which one forecast is beating another. A positive or negative slope indicates a change in the proportion of wins of one or other of the forecast systems relative to the prior win proportion. For the first few years in each of the plots in Fig. 2a–e, the slope changes randomly, reflecting the small number of comparisons and the role played by chance for such small numbers. As the number of forecast comparisons increases, the slopes tend toward zero indicating that the forecast comparison has stabilised in the sense of reflecting the relative levels of skill of the two forecast systems. However, the skill levels of forecast systems may change through time, reflecting changes in the systems themselves^15^.

The results for 4 of the 5 bias-correction methods in Fig. 2 panels a–e show that the CGCMs are similar (cm3, cm4i, nemo, and seas5) or a bit worse (remaining models) than the regression model, consistent with DelSole and Tippett^15^. For the *unfair* method there is no difference between the CGCMs and the regression forecasts (scores sit mostly in the ‘chance’ region in Fig. 2), but this method uses the inappropriate climatology in the test period to derive forecast anomalies.

The results for the fair methods (*fair*, *fair*-*sliding*, *fair*-*all*) in Fig. 2 panels a–e are broadly similar, though the dynamical models ultimately do better using *fair*-*sliding* and *fair*-*all* than *fair*. This is understandable in that the *fair* reference climatology is fixed in the training period and is further separated in time from each new forecast start in the testing period as the forecasts march through the testing period in the random walk score. The reference climatologies for *fair*-*sliding* and *fair*-*all* move along behind each new forecast in the testing period and so do not suffer the disadvantage of using a reference climatology increasingly removed from the testing period.

The results shown here in Fig. 2 at lead time 3(a–e) are broadly repeated at all of the lead times (1 to 11 months) tested. The final value of the random walk skill score (after completing all forecast comparisons in the test period) is shown as a function of lead time in Fig. 2 panels f–j and indicates two broad groups of models. One set (cm3, cm4i, nemo, gmao, seas5) has similar skill to the regression model regardless of whether the *fair* or *unfair* method is used. The seas5 model is the best performing CGCM relative to the regression model at short leads, but its skill is indistinguishable from linear regresson by 6 months lead. The other set of models (aer04, cola, florA, florB) are generally a bit worse than the linear regression model unless the bias-correction method for the models is the *unfair* one. The results for the comparison with a linear regression model here are broadly similar if using a logistic regression model instead (see Supplementary Note 3).

One important feature of the results in panels f–j in Fig. 2 is that the rankings of the models relative to one another changes depending on the method used to form the anomalies : *biased*, *unfair*, *fair*, *fair-sliding*, or *fair-all*. The relative model rankings at lead 6 (the longest lead for which we have all models) is shown for each of the bias correction methods. The changes in ranking between *unfair* and *fair* methods are striking. For example, florA and aer04 are among the most skillful models for *unfair* and among the least skillful for *fair*. The relative rankings change much less among the different variants of the *fair* method.

## Comparison of fair and unfair method

**Fig. 3:**
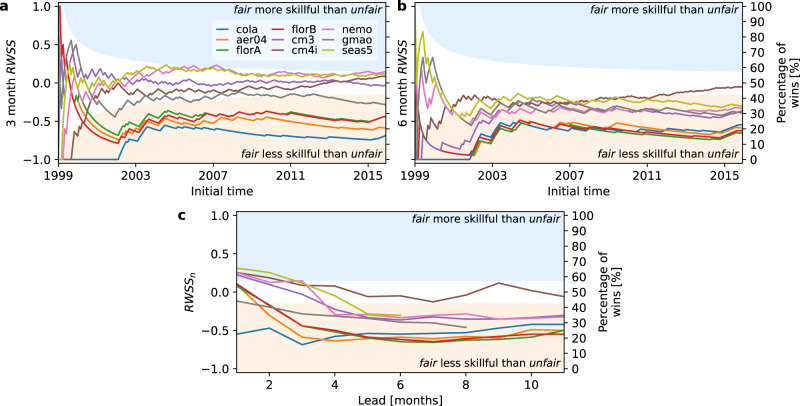
Comparison of El Niño Southern Oscillation (ENSO) skill for *fair* and *unfair* methods. Random walk skill score (*RWSS*) for lead 3 (**a**), lead 6 (**b**), and *RWSS*_*n*_ as a function of lead time (**c**).

Thus far we have seen that the dynamical models perform better relative to simple regression models when using *unfair* than *fair*. In this section we perform a direct comparison between *unfair* and *fair* methods for the same forecast model bias corrected in the two different ways. A direct comparison using *fair* and *unfair* methods for all dynamical models is shown in Fig. 3. At 3 months lead (panel a) the models again fall into two broad classes; one set (cm3, cm4i, nemo, seas5) that are largely indifferent between *fair* and *unfair*, and another set (aer04, cola, florA, florB) where the *unfair* method is more skillful than *fair*. By lead 6 (panel b) almost all the models (excepting only cm4i) are now in the zone where *unfair* is more skillful than *fair*. For the second group of models above the advantage is striking where the *unfair* method wins 80% of forecast comparisons to *fair’s* 20%.

As a function of lead time, the random walk skill score, *RWSS*_*n*_, (panel c in Fig. 3) is ambiguous in selecting the more skillful method at very short lead (0, 1 months). There is little advantage to the *unfair* method close to initialisation, but as lead time increases the *unfair* method progressively dominates *fair*. The main difference between models is how quickly this occurs as a function of lead time, with one group of models (aer04, florA, florB) where *unfair* dominates *fair* by about lead 3, and another group (cm3, nemo, gmao, seas5) where this occurs by about lead 4 or 5. The exceptions to the progressive dominance of *unfair* with lead time are cola, where a change in initial condition error between training and testing periods dominates the performance at low lead times (see Supplementary Note 2), and cm4i, which remains indifferent between *fair* and *unfair* over all lead times.

The cross-validated form of the unfair method, *unfair*-*cv* performs similarly to the pure *unfair* method in dominating *fair* forecasts (see Supplementary Note 6). A visualisation of the advantages of the unfair methods is given in Box 3. The reasons for their advantages are related to the biases in the models and are investigated next.

Box 3 Cross validation and unfair hindcasts To visualise the advantage that the *unfair* hindcasts have over *fair* hindcasts, we show a time series of Niño3.4 at lead 6 for *fair*, *unfair*, and observations in Fig. Box 3 panel **a**. The forecast anomalies for the *fair* method are (on average) about a degree too warm, whereas the *unfair* method anomalies are much closer to observations. The distributions of the forecast anomalies at lead 0^*^ (Fig. Box 3 panel **b**) are well centred for *fair* and *unfair* forecasts, but by lead 6 (Fig. Box 3 panel **c**) the *fair* forecast anomalies are generally offset. The consequences of the *fair* anomaly offset for the skill of the *fair* ENSO forecasts are quite drastic because the anomaly shift of about 1 ^∘^C is twice the magnitude of the Niño3.4 anomaly (0.5 ^∘^C) used to define an ENSO event.

Some have argued that the *unfair* method can be made fairer by carrying out a form of cross-validation when deriving the reference climatology in the testing period^21,27,45^. In the cross-validated form of the unfair method, *unfair*-*cv*, whenever an anomaly is calculated, a window of forecasts centred about the anomaly is removed from the set of forecasts used to compute the baseline forecast climatology. As the data comprising the forecast climatology contains significant autocorrelation, it is argued that a span of typically about 3 years needs to be excluded in the anomaly window^45^. We have tested this with odd-length windows from 1 to 7 years width. The results are not very sensitive to the width selected and so we chose a 7-year window to maximise the effect of cross validating. The *unfair*-*cv* method is just as skillful as the *unfair* method in outperforming the fair method (see Supplementary Fig. 6). By leaving a year out of the reference climatology (or even 3, or 5, or 7 as we show here), the reference climatology still benefits from having all of its years taken from the test period. Years in the test period are initialised in the background state that applies to the forecasts, and the anomalies formed from that climatology are reasonably well centred (dotted lines in Fig. Box 3 panels **b** and **c**). Leaving some data out of that climatology does not de-centre the anomalies anywhere near as much as using the training period (solid lines). To show how little difference the cross-validation makes to the *unfair* method, the *unfair* and *unfair*-*cv* anomalies are plotted together, and practically lie on top of one another in both the time series (Fig. Box 3 panel **a**) and pdfs in Fig. Box 3 panels **b** and **c**. The near equivalence of the *unfair* and *unfair*-*cv* anomalies explains why the skill determinations are nearly identical for these two methods. Since the cross-validated climatology, *unfair*-*cv*, makes almost the same transgressions (of test data) as the *unfair* method and retains most of the benefits of those transgressions, we do not regard it as a valid way to make the *unfair* method fairer.

Fig. Box 3: Observed and model forecast Niño3.4 anomalies. In panel **a** the black line shows observed Niño3.4 anomalies in the testing period. The red lines are for the florB ensemble mean Niño3.4 anomalies at lead time 6 for anomalies calculated by the methods *fair*, *unfair*, and *unfair*-*cv*. The *unfair*-*cv* anomalies are calculated removing a 7-year cross-validation window. The probability density distribution of the anomalies is shown for each dynamical model over all months at lead time 0^*^ (panel **b**) and lead 6 months (panel **c**) in the testing period. The anomalies have been calculated according to the anomaly methods, *fair* (solid lines), *unfair* (dashed), and *unfair*-*cv* (dotted).

## Why are unfair hindcasts advantaged?

**Fig. 4:**
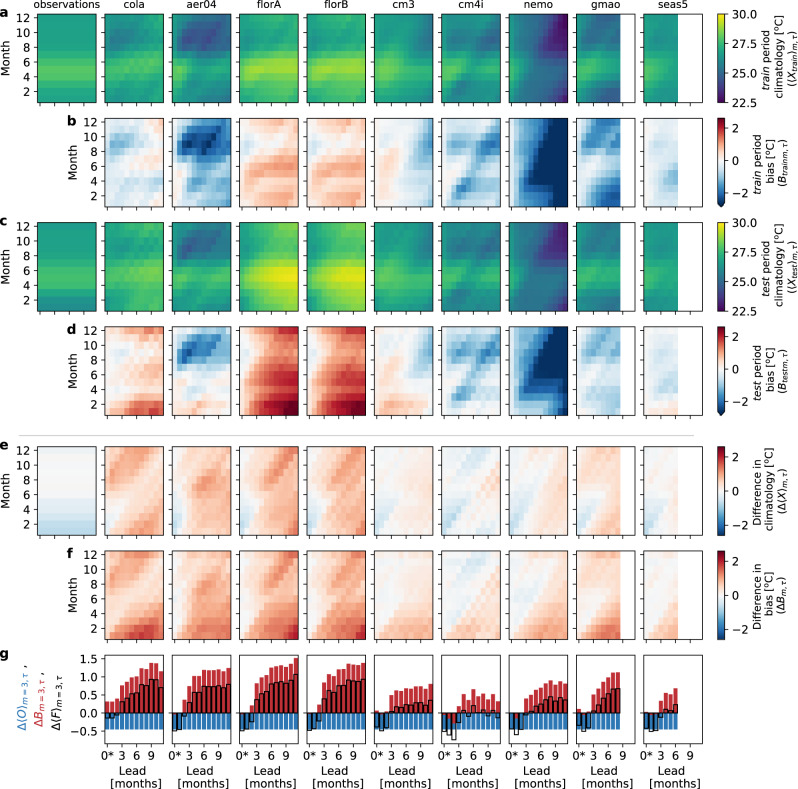
Observed and model climatologies and biases. Row **a** is the observed climatology, ${\langle {O}_{{\rm{train}}}\rangle }_{m,\tau }$, and set of model forecast climatologies, ${\langle {F}_{{\rm{train}}}\rangle }_{m,\tau }$, for the training period (as functions of calendar month and lead time). Row **b** shows the training period biases, ${{B}_{{\rm{train}}}}_{m,\tau }$, which are the differences between the model and observed climatologies in the training period, ${\langle {F}_{{\rm{train}}}\rangle }_{m,\tau }-{\langle {O}_{{\rm{train}}}\rangle }_{m,\tau }$. Row **c** is the observed climatology, ${\langle {O}_{{\rm{test}}}\rangle }_{m,\tau }$, and set of model climatologies, ${\langle {F}_{{\rm{test}}}\rangle }_{m,\tau }$, for the testing period. Row **d** shows the testing period biases, ${{B}_{{\rm{test}}}}_{m,\tau }$, which are the differences between the model and observed climatologies in the testing period, ${\langle {F}_{{\rm{test}}}\rangle }_{m,\tau }-{\langle {O}_{{\rm{test}}}\rangle }_{m,\tau }$. Row **e** is the difference in climatologies between the training and testing periods for observations, ${{\Delta }}{\langle O\rangle }_{m,\tau }={\langle {O}_{{\rm{test}}}\rangle }_{m,\tau }-{\langle {O}_{{\rm{train}}}\rangle }_{m,\tau }$ (first column) and models, ${{\Delta }}{\langle F\rangle }_{m,\tau }={\langle {F}_{{\rm{test}}}\rangle }_{m,\tau }-{\langle {F}_{{\rm{train}}}\rangle }_{m,\tau }$ (remaining columns). Row **f** is the difference between the model biases in the training and testing periods, ${{\Delta }}{B}_{m,\tau }={{B}_{{\rm{test}}}}_{m,\tau }-{{B}_{{\rm{train}}}}_{m,\tau }$. Row **g** is a bar plot for the month of March (month 3 above) of the observed change in climate, Δ〈*O*〉_March,*τ*_, (blue bars), the change in model biases, Δ*B*_March,*τ*_, (red bars), and the sum of these, which is the change in model climatology, Δ〈*F*〉_March,*τ*_, (black outlines).

To understand why the *unfair* method benefits so much by using a reference climatology from the test period, one needs to assess the climatologies formed in the training and test period and how they shape the forecast anomalies in the test period. The model forecast climatologies are typically broken down by calendar month and lead time as shown in Fig. 4. The training period climatology is in row a and the test period climatology is in row c. The difference in these model climatologies, Δ〈*F*〉_*m*,*τ*_ is shown in row e. Any difference between these reference climatologies leads to different outcomes for the *fair* and *unfair* methods. We show why this is so and examine the contributions to this difference.

The model forecast anomalies are generated for the *fair* and *unfair* methods by subtracting the reference climatologies in the training (row a) and testing (row c) periods respectively from the forecast values. For the *unfair* method, since the reference climatology covers the period tested, the climate state and model biases are estimated exactly and removed such that the mean of the forecast anomalies (as functions of month and lead) are zero. This means that the *unfair* forecast anomalies are all well centred (see panels b and c in Fig. Box 3). However, for the *fair* method, any shift in climate state or change in model bias from the training to testing period is retained in the forecast anomalies, which are offset by the amounts shown in row e of Fig. 4. These offsets can be up to 1.5 ^∘^C for some months and leads and detract from skill of the *fair* forecasts.

There are two major contributors to the differences in reference climatology between the training and testing period:

1. the nonstationarity or shift in climate between the periods (a change in the observed climatology)
2. the change in model bias from training period to testing period

There will also be differences in mean climatology due to the limited sample sizes of the climatological distributions here, which are hard to quantify.

### Climate shift

The climate shift contribution, Δ〈*O*〉_*m*,*τ*_, is represented by the difference between the observed climatologies in the training and testing period in the first column of row e. There is actually a slight cooling in Niño3.4 from the training to test period. This cooling is consistent with the change in phase of the Pacific Decadal Oscillation^24^ from a predominatly warm phase (El Niño dominated) in the training period to a predominantly cold phase (La Niña dominated) in the testing period^25^. The change in model climate from the training to testing period, Δ〈*F*〉_*m*,*τ*_, is shown in the remaining columns of row e. At short lead times, the cooling climate shift in observations is communicated to the forecast climatologies because each of the forecasts making up the forecast climatologies are initialised about the set of observations in these periods. At longer lead times, the model forecast climatologies are increasingly too warm (e.g., Fig. Box 3 panel c), tending to overheat El Niño’s and underplaying the colder La Niña events.

### Change in model bias

The bias in the models depends on the climate state^21^, and since there is a shift in climate state between the periods, there is a change in the profile of model bias between the periods. The model bias is the difference between the forecast climatology and observed climatology and is shown for the training and test periods in rows b and d of Fig. 4. The biases vary between models, with mostly cold bias in aer04, cm4i, nemo, and gmao, mixed bias in cola, cm3, and seas5, and warm biases in the flor models. The change in the profile of model bias between the periods is evident with most of the models showing warmer biases in the testing period than the training period. The difference in bias between the periods, Δ*B*_*m*,*τ*_, is shown in row f. The warming of the biases is evident with the preponderance of positive values in this row. The models fall into roughly two groups. A high Δ*B*_*m*,*τ*_ group (cola, aer04, florA, florB) has relatively large change in bias across the periods, and a low Δ*B*_*m*,*τ*_ group (cm3, cm4i, nemo, seas5) has smaller change in bias. The gmao model is somewhat intermediate between the two groups. The high Δ*B*_*m*,*τ*_ models warm substantially in the testing period relative to the training period, whereas the observations cool, which results in a larger change in bias. For all models here, the magnitude of Δ*B*_*m*,*τ*_ increases with lead time, *τ*, as expected.

### Relative contributions of shift in climatology and change in bias

The forecast anomalies for the *fair* method have a mean offset relative to those for the *unfair* method given by the change in model climatology from training to testing period, Δ〈*F*〉_*m*,*τ*_, in row e of Fig. 4. This offset has contributions from both the climate shift and the model bias change, so that the values for the models in row e are the sum of the bias changes in row f and the climate shift for observations at the beginning of row e. At short lead times (<3 months) the change in model bias, Δ*B*_*m*,*τ*_, is still small and the change in model climatology, Δ〈*F*〉_*m*,*τ*_, is dominated by the climate shift in observations, Δ〈*O*〉_*m*,*τ*_. This is evident in the cooling (blue shades) in model climatology in months 1–5 (Jan–May) at short leads in row e. At longer leads (>3 months), the change in climatology is increasingly positive for all models, and it is clear that most of the contribution to Δ〈*F*〉_*m*,*τ*_ then comes from the model warm bias shift, Δ*B*_*m*,*τ*_. This point can be illustrated by considering these changes for a single calendar month (March) in row g. The climate shift in observations, Δ〈*O*〉_March,*τ*_ (blue bars) is the same at all lead times of course, whereas the change in model bias, Δ*B*_March,*τ*_, tends to increase with lead time (red bars). The sum of these is the change in model climatology, Δ〈*F*〉_March,*τ*_, which is nearly equal to Δ〈*O*〉_March,*τ*_ for leads < 3, and then is increasingly positive with lead as the model change in bias tends to dominate the sum.

A hypothetical model with no biases (or at least no change in bias, Δ*B*_*m*,*τ*_ = 0) would still exhibit sensitivity to the choice of *fair* or *unfair* methods whenever there is any change in the observed climate. Conversely, a model for which there is no change in climatology from training to testing period (Δ〈*F*〉_*m*,*τ*_ = 0) would be insensitive to choice of *fair* or *unfair*, but it would also mean that the model hasn’t accurately captured any observed change in climate (Δ〈*O*〉_*m*,*τ*_) between the two periods. The models here that have lowest sensitivity to *fair* or *unfair* (cm3, cm4i, seas5) partly do so because their warming biases in months 1–5 are nearly opposite to the observed cooling in these months, and partly cancel to produce lower changes in forecast climatology. This cancellation is particularly apparent in the month of March for cm4i, which has near zero change in climatology from lead 3 onwards (Fig. 4 row g).

## ENSO onset

Thus far we have treated every month’s forecast as a forecast comparison point in the assessment of ENSO skill, irrespective of what state ENSO is in. That means that we include forecasts of many times when ENSO is in its (relatively less interesting) neutral state (about 40–50% of the time, depending on the data used to measure it). For this full set of forecasts we find that the dynamical models are generally no better than simple regression models. One might expect that the dynamical models come into their own when ENSO itself changes state, such as during the onset and decay of El Niño and La Niña events. We now restrict our evaluation of the ENSO forecasts to just those forecasts relating to onset.

For onset we discuss hindcasts using the *fair* method here, since this is more indicative of actual forecast skill. We use the onset case study to further highlight variability in apparent skill that results from different hindcast periods. This is explored by considering the standard case where the reference period is 1982–1998 and the testing period is 1999–2016, and a switched case where the reference period is 1999–2016 and the testing period is 1982–1998. In the switched case, the change in bias with lead time of the model climatologies is in the opposite sense to the standard case. The models tend to cool with increasing lead time in the switched case, and the change in observed climatology is now a warming. That is, rows e and f in Fig. 4 are of the opposite sign for the switched case.

**Fig. 5:**
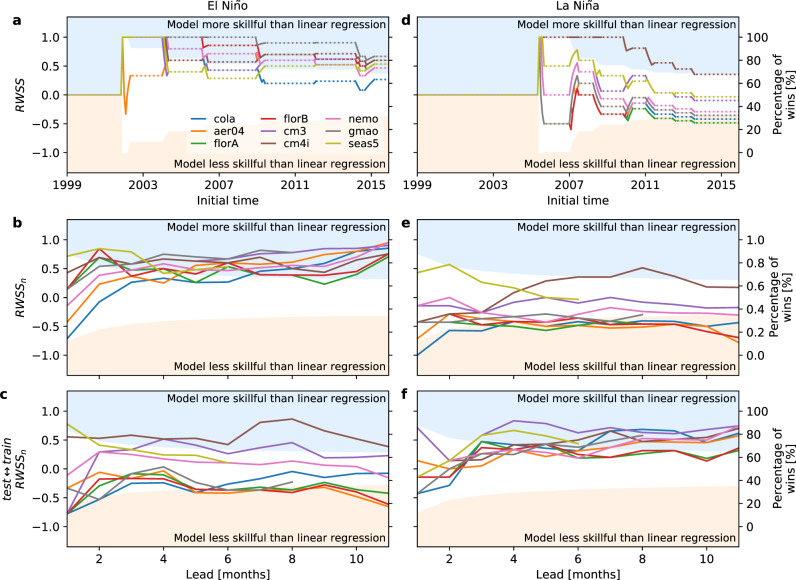
Skill scores for El Niño and La Niña onset using the *fair* method. Random walk skill score (*RWSS*) at 6 months lead relative to linear regression for El Niño and La Niña onset (**a** and **d**). The dashed lines in the panel represent forecast comparison periods that do not correspond to `onset' comparisons. The final random walk skill score, *RWSS*_*n*_, is shown as a function of lead time for El Niño and La Niña onset in panels **b** and **e**. Panels **c** and **f** are the same as panels **b** and **e**, except that the training and testing periods have been switched.

The skill scores for El Niño onset at 6 months lead relative to the linear regression model are shown in Fig. 5 panels a–c. The random walk score only changes when there is an onset forecast, and there are only about half a dozen onset forecasts during the testing period. The final random walk scores in panel b indicate that the dynamical models are more skillful than the regression model at all leads. In this standard case, the tendency of the dynamical models to develop warm biases with increasing lead time in the testing period works in their favour. El Niño onset involves a warming of sea surface temperature with lead time from the pre-onset state. The tendency of the dynamical models to warm with lead time from a given initial state in the standard testing period is fortuitous for warm event onset forecasts. We can test this by reversing the training and testing periods, and repeating the onset forecast comparisons on the first half of the record (1982–1998). The results for this switched case are shown in panel c. In this case the dynamical models now only do about the same as linear regression at all leads. In other words, there is a dramatic change in relative performance just by switching training and testing periods. In the switched case, the models now generally have a cold bias with increasing lead time in the switched testing period, which works against their forecasts of the warming associated with onset.

We now turn to forecasts of La Niña onset. Since La Niña onset represents a cooling of sea surface temperatures with lead time, we might expect the opposite behaviour here to the El Niño onset case. That is, we’d expect the dynamical models to be about the same (or a bit worse) than the linear regression model for La Niña onset at all leads for the original training and testing periods, because the models’ increasing warm bias with lead works against onset of a cold event. Moreover, we’d expect the dynamical models to be better than the regression model at most leads for the switched periods, where the model cold bias with lead assists their forecasts of cold event onset. That is precisely what we do see in Fig. 5 panels e and f for the original periods and switched periods.

We now have the conundrum that if we choose our original training and testing periods, the dynamical models are better/worse for El Niño/La Niña onset than regression models, and if we switch the periods the reverse is true. This result highlights the interaction between model biases, the underlying ENSO time series, and the nature of the event tested. Where the sign of an event onset (warming/cooling) lines up with lead-dependent change in climatological bias in the dynamical models, it is possible to substantially enhance (when in the same direction) or degrade (when in the opposite direction) apparent skill for onset. To guard against this, we would ideally have many periods over which to test onset, though that is not possible with our limited observational record. As such, it is important to be aware how the model biases change from training to testing periods, and how they interact with the event tested. Otherwise, conclusions about the relative skill for onset of El Niño and La Niña may substantially reflect the periods chosen.

The sensitivity of skill results to the choice of period applies to *fair* forecasts, because they retain lead-time-dependent change in bias across periods. For the *unfair* method, these biases are removed and the sensitivity of results to choice of period is attenuated (see Supplementary Note 7).

## Impact of bias correction on skill

The manner in which bias correction methods can affect hindcast skill can be understood from the set of hindcasts assessed here. Hindcast skill will typically be inflated if the reference period used to form ENSO anomalies overlaps with the testing period in which hindcast skill is assessed (the *unfair* method). When the reference period does not overlap the testing period (the *fair* method), then there will inevitably be some difference between the climatology of forecasts made in the testing and reference periods. This difference in forecast climatology changes the skill of *fair* forecasts relative to *unfair* forecasts. The difference in forecast climatology results from any climate shift in observations from the reference to the testing period, and from changes in model bias (the difference between the model and observed climatology) between the reference and testing period. At short lead times (1 or 2 months), the change in model bias is typically small, and the shift in observations is the dominant contributor to the change in forecast climatology here. At lead times beyond 3 months, the change in model climatological bias is the dominant contributor to the change in model forecast climatology between the reference and testing periods, and is thus also the main contributor to degradation of skill of *fair* forecasts relative to *unfair* forecasts.

The magnitude of the change in bias (between the reference and testing periods) increases with lead time for all of the models here. That means that *unfair* bias correction methods progressively inflate skill (relative to *fair* methods) as lead time increases. This is true for even the best performing models here (cm3, cm4i, nemo, seas5). The best performing models have low initial condition error and low change in bias between the reference and testing periods, but their change in bias also grows with lead time such that *unfair* forecasts progressively beat *fair* forecasts.

As such, assessments of ENSO seasonal hindcast skill can be sensitive to the method used to define the reference climatology and associated forecast anomalies. Any method that uses the forecast testing period as a basis for the reference climatology (the *unfair* method here) may reflect some amount of artificial skill not realisable in real forecasts where the future is unknown. *unfair* method forecasts remove both the contributions of climate drift^26^ and change in model bias from the forecast anomalies. They provide an indication of what skill could be if the model biases were eliminated in the forecast models. For any *fair* forecast, the *changes* in model bias from the period in which the reference climatology is formed to the period in which the forecast is evaluated, constitute the dominant enduring contribution to skill degradation for the dynamical model forecasts tested here.

## Lessons for forecast skill assessment

Progress towards the goal of improving climate forecasts needs to be quantified by fair skill assessments. At present, seasonal forecasts are typically assessed by the institutions that produced them and are not easily comparable across institutions. In many papers and hindcast/forecast archives the bias correction method is not clearly stated or not consistent across models^10^, rendering any subsequent skill assessment or comparison of dubious use. To remedy this, skill assessments should be performed by open communities on open platforms. Model groups need to provide the raw hindcast outputs so that all models can be subject to identical bias-corrections and so be meaningfully compared.

The forecast assessment literature sometimes implies that forecast anomalies calculated using the *unfair* method can be made fairer by performing a form of cross-validation in constructing the reference climatology. The WMO guidelines for forecast assessment state that ‘It is generally considered to be best practice to calculate the bias correction in a ‘cross-validated’ manner where the particular forecast to be corrected does not contribute to the forecast average’^27^. Our results with the NMME and C3S model forecasts show that cross-validation does little to change the results obtained using the pure *unfair* method. Applying cross-validation to the reference climatology formed from the testing period still removes most of the bias from the model hindcasts, thereby retaining most of the artificial skill in the hindcast assessment.

We have emphasised the importance of processing and comparing hindcasts in the same way. However, even if all hindcasts are compared using the same *unfair* method, that does not uniquely determine how different forecasts will rank relative to one another using a different (*fair*) method. The relative rankings of models change depending on the nature of the biases in the different forecast systems. For example, some of the models tested here (cm3, cm4i, seas5) have relatively low bias changes across different reference periods, and thus their skill scores are not too sensitive to the way the model biases are processed. The other models tested have larger bias changes across different climate periods and perform much better when their forecasts are processed with the *unfair* method than with the *fair* method. This means that the relative model rankings change depending on how they are bias corrected.

The prevalence of *unfair*-based methods in hindcast assessments is widespread and perhaps understandable. Resources to develop long hindcast databases are often lacking, and the user faced with limited hindcast data needs to use all hindcast runs to get a more stable estimate of model biases^28^. However, the use of *unfair*-based methods in hindcast assessment is sometimes rationalised on the basis that if all models are processed similarly, then that maintains a level-playing field to assess models relative to one another^29,30^. Unfortunately, that is not the case, as we showed that the relative rankings of models can change in going from an *unfair* to *fair*-based method.

The use of *unfair*-based methods in hindcast assessment continues to be recommended for use in climate forecast guidance and standards documents^21,27,28^. The pitfalls around this may be transparent in the climate forecast community. However, those who use hindcast databases to assess forecast utility for applications are often not aware that their assessments could be based on artificial skill (if the hindcasts have been processed using *unfair*-based methods). For example, much of the assessed value of seasonal climate forecasts for Australian agriculture is based on hindcasts processed using *unfair* methods^31^, which therefore contain artificial skill. This can be misleading for farmers who must make tactical decisions using real forecasts, not hindcasts, and thus are likely to have lower forecast skill than expected.

## Path ahead

When the assessment of model skill is fair, the skill of the NMME and C3S dynamical forecast models for ENSO is generally not demonstrably better than simple regression models. We tested whether dynamical models would perform better if forecast comparisons were restricted to just the more dynamically challenging onset aspects of El Niño and La Niña events. We drew no clear conclusions in this regard because the results for onset are critically sensitive to the direction of the change in forecast climatological bias in the models from calibration to testing periods. If this change in bias is toward warming, then warm event (El Niño) onset skill is high, and conversely if toward cooling, then cold event (La Niña) onset skill is high. However, since the change in climatological bias in the models is a function of the periods assessed (warm to cold, or cold to warm), then so too is the perception of relative skill for El Niño and La Niña onset.

From the comparison of hindcast skill of *fair* and *unfair* methods here, we can deduce that the skill of actual forecasts of ENSO will be limited by the growth with lead time of climate-state-dependent biases in the dynamical models. A partial solution to this problem is to use more sophisticated bias-reduction schemes that take into account the state of ENSO^32,33^, the underlying trends in the observed and model hindcast climatologies^33,34^, and the extension to multivariate processes^35^. That will reduce, but not eliminate, the effects of climate-state-dependent biases. The long-term goal remains to diagnose the reasons for climatological biases and to reduce their magnitudes and effects on forecast skill.

## Methods

### Observational data

The observations used in the analysis cover sea surface temperature (sst) in the Niño3.4 region (5^∘^S–5^∘^N; 170^∘^W–120^∘^W). The sst data used are from OISST v2^36^. All analysis was repeated with HadISST^37^, which does alter some of the details of the results, but not the more general conclusions drawn. We define El Niño/La Niña events when the 3-month running mean of Niño3.4 anomalies is above or below ± 0. 5^ ∘^C, respectively. The general results presented here are unchanged if Niño3 or Niño4 are used instead of Niño3.4.

### Dynamical models

The models used here are from the North American Multimodel Ensemble (NMME)^16^ and the Copernicus Climate Change Service (C3S)^38^. The advantage of the NMME archive is that it contains a rich set of runs from many CGCMs over a relatively long period from 1982 to the present. Another advantage is that the NMME archive has been the source of a number of prior ENSO skill assessments^15,16,29,30,39–41^. We selected models from these two archives that satisfied the criteria of: raw (uncorrected) hindcast data available, lead-time forecasts out to at least 6 months, at least 10 ensemble members per forecast, and hindcasts/forecasts span the period 1982–2015. The subset of models satisfying these criteria are CMC1-CanCM3 (cm3), CanCM4i (cm4i), COLA-RSMAS-CCSM4 (cola), GEM-NEMO (nemo), GFDL-CM2.1-aer04 (aer04), GFDL-CM2.5-FLOR-A (florA), GFDL-CM2.5-FLOR-B (florB), NASA-GMAO-062012 (gmao), and CDS-C3S-ECMWF-SEAS5 (seas5). The only qualifying model from C3S is seas5. All other models are from NMME. All models contain leads to 12 months, except GMAO to 9 months, and seas5 to 6 months. The cola model has a documented discontinuity in initial conditions (see Supplementary Note 2).

Though we refer to the NMME outputs as ‘forecasts’, the archive contains a mix of hindcasts and forecasts. The hindcasts all commence in 1982 and switch over to forecasts in 2011 for the CanCM models and GFDL-aer04, and in 2014 for the remaining models. Some of the hindcasts commence on the first of each month, whereas forecasts may commence after the first of each month^16^. The model forecasts have exchangable members (with similar statistical properties and skill), except for COLA-RSMAS-CCSM4, which employs a lagged atmosphere^15^. We use the first 10 ensemble members from each model. As the forecasts may start after the first day of the first calendar month, the first calendar month in principle contains some assimilated observations and some period of pure forecast. This month thus represents in part the initial state and in part some forecast, but is not strictly either. As such, we denote the lead time for the calendar month in which the forecast commences as lead 0^*^, where the * indicates that the lead is in fact not well defined here. The forecasts for the second calendar month span leads of roughly half a month to one and a half months, and thus have an average lead time of 1 month. We designate these as lead 1 (month), and so on for subsequent lead times.

### Regression models

We use two simple regression models as skill baselines. The first follows DelSole and Tippett^15^ in using ordinary linear regression on observations of Niño3.4. The regression provides a fit between Niño3.4 values in the calendar month, *m*, where the forecast is initiated, ${\mathrm{Ni}}{\tilde{\mathrm{{n}}}{\mathrm{o}}}_{m}$, and values in the target calendar month at lead time *τ* being forecast, ${\mathrm{Ni}}{\tilde{\mathrm{n}}}{\mathrm{o}}_{m+\tau }$. The forecast value of Niño3.4 then uses the regression coefficients, ${\hat{a}}_{m,\tau },{\hat{b}}_{m,\tau }$, from these fits to predict lead values on the basis of the current initial value as ${\mathrm{N}}{\hat{{\mathrm{i}}}}{\tilde{{\mathrm{n}}}}{\mathrm{o}}_{m+\tau }={\hat{a}}_{m,\tau }{\mathrm{Ni}}{\tilde{{\mathrm{n}}}}{\mathrm{o}}_{m}+{\hat{b}}_{m,\tau }$. The second regression model is an ordered logistic regression^23^ based on the categories La Niña, neutral, El Niño from Niño3.4 values.

### Random walk skill

The method used to compare forecasts here is the random walk sign test introduced by DelSole and Tippett^15^. This test provides an intuitive way to visualise the difference between two forecasts, *f*_1_, and a reference forecast *f*_*r*_, with an assessment of confidence that does not depend on particular distributional assumptions about the forecast errors. A sequence of instances, *i*, of *n* matched forecasts, ${{f}_{1}}_{i}$ and ${{f}_{r}}_{i}$ are compared at a given lead time. The sequential comparisons are ‘walked’ with a count, *RW*_*i*_ incrementing/decrementing by one whenever the squared error of the model being tested, ${({{f}_{1}}_{i}-{o}_{i})}^{2}$, is greater/less than that of the reference model, ${({{f}_{r}}_{i}-{o}_{i})}^{2}$. The scoring is binary and does not scale the score by the relative magnitude of the forecast errors, since that form of scoring is more likely to be biased when the forecasts tested are not independent^15,42^. Serial correlation among the forecasts is an issue for many skill scores, including this one^15^. Binary scoring will reward a model that is closer to observations more often, even if it does sometimes have large forecast errors. The binary count traces a random walk, which can be compared with an envelope encompassing the range of counts that would be observed 95% of the time by chance under independent Bernoulli trials for *p* = 1/2. The envelope expressing this range is approximated by $1.96(-\sqrt{{n}_{i}},\sqrt{{n}_{i}})$^15^. We develop and apply here a standardised version of the random walk as a random walk skill score: *RWSS*_*i*_ = (1/*i*)*RW*_*i*_, *i* = [1, *n*] forecast comparisons. The chance envelope for this score is also (1/*i*) times the RW envelope. For any comparison, *i*, the *RWSS* sits between 1 (reflecting a forecast that beats the reference every time for forecast comparisons [1, *i*]) and −1 (reflecting a forecast that loses to the reference every time).

To compare results for the random walk sign test as a function of lead time, at each lead time (for lead months 1–11) we take the value of *RWSS*_*i*_ for the last forecast comparison (*i* = *n*). This value, *RWSS*_*n*_, represents our final estimate of the relative skill of the two models tested at each lead time. The score for *RWSS*_*n*_ ranges from 1 (always beats reference) through 0 (model ties with reference) to −1 (always loses to reference). The plots of *RWSS*_*n*_ also include the 95% chance envelope, where the bounds of the envelope are those that pertain to the last forecast comparison for *RWSS*.

### Categorical random walk

The random walk score described above uses a distance test (squared error) to reward the model lying closest in distance to the observations at each forecast comparison. In principle, any test can be used for each forecast comparison in the random walk framework. The random walk score can easily be adapted to categorical forecasts, and we do that here for use with the categorically based logistic regression forecasts. In the categorical forecast tests, all forecasts are translated in terms of ENSO category (El Niño, neutral, La Niña). If one model forecasts the correct category but the other model doesn’t, then the correct model scores one for that test. If the models forecast the same category (right or wrong), no score increment is made for that forecast comparison. No increment to the score is made if both models forecast the wrong category. In particular, no credit is given if one model lands closer to the observed category than the other model if neither are in the correct category. Thus, with the categorical random walk scores, there will be many ‘draws’ where no increment is made, unlike the case for the distance-based random walk score, where draws are unusual. In other respects though, the categorical random walk scores are identical to those described above for the conventional distance-based random walk skill scores.

### Bias correction and anomaly calculation

We use several different methods to generate anomalies for the model forecasts. The methods are represented schematically in Fig. Box 2. The schematic shows a time series of the observed variable at the top, with the time series divided in half to give a training period and a test period. The training period is typically reserved for generation of a climatology to serve as a baseline reference for the forecasts, and the test period is used to test the anomalised forecasts. For each anomaly calculation method, the calculation is performed by developing a reference climatology, *r*, for the forecast as a function of both calendar month, *m*, and lead time, *τ*, denoted 〈*r*〉_*m*,*τ*_ where 〈 ⋅ 〉 indicates temporal averaging. The reference climatology is then subtracted from the forecasts, *F*, (as a function of *m* and *τ*) to produce an anomaly forecast, *f*. All results in the paper were repeated with the training and testing periods reversed (train on testing period, test on training period). For the analysis of ENSO onset the ordering matters and both sets of results (standard and switched) are presented. For all other forecast assessments here the results are qualitatively similar either way, and so only results for the standard periods are presented.

### Biased

The first method used here we refer to as the ‘*biased*’ method as it retains model bias in forming the anomalies. The reference climatology is built from observations in the training period, *O*_train_, (Fig. Box 2, purple box), $\langle r\rangle ={\langle {O}_{{\rm{train}}}\rangle }_{m}$. Then the *biased* anomaly forecast is given by ${f}^{{\rm{obs}},{\rm{train}}}=F-{\langle {O}_{{\rm{train}}}\rangle }_{m}$, where the superscript ^obs,train^ indicates that the anomalies are based on a reference climatology from observations in the training period.

### Unfair

In the second method, the reference climatology is based (inappropriately) on model forecasts in the test period (Fig. Box 2,pink box), $\langle r\rangle ={\langle {F}_{{\rm{test}}}\rangle }_{m,\tau }$. Since this is unfair to real-time forecasts, we refer to this as the *unfair* method. *unfair* forecasts are given by ${f}^{{\rm{fcst}},{\rm{test}}}=F-{\langle {F}_{{\rm{test}}}\rangle }_{m,\tau }$. In practice, the model reference climatology for the *unfair* method is usually calculated from the entire hindcast period covering both the training and testing periods in the schematic in Fig. Box 2; $\langle r\rangle ={\langle {F}_{{\rm{train}}+{\rm{test}}}\rangle }_{m,\tau }$. This yields very similar results to using the test period only. We restrict the definition of *unfair* to the more restricted test period here so that the length of the reference climatology is equivalent in both the *fair* and *unfair* methods, and only the period varies.

### Unfair cross-validated

In the *unfair*-*cv* method the reference climatology in the test period is calculated in a cross-validated sense, where a segment of the set of forecasts is removed from *F*_test_ in the period surrounding the time at which each *f*^fcst,test^ is calculated. Here, we remove the year in which the anomaly is calculated and an additonal set of years either side of that year. We test variants of this with 1, 3, 5, and 7 years total removed in a centred window on the year in which the anomaly is calculated. This cross-validated *unfair* method is denoted as *unfair*-*cv* and is represented by the dashed red lines in Fig. Box 2. The results for *unfair-cv* are not very sensitive to the window length used here, so we show results for the 7-year window to maximise any potential affect of cross-validation.

### Fair

For this method the reference forecast climatology is based appropriately in the training period. We refer to this method as *fair*. The *fair* reference climatology, $\langle r\rangle ={\langle {F}_{{\rm{train}}}\rangle }_{m,\tau }$ corresponds to the green box in Fig. Box 2. The *fair* anomaly forecasts are ${f}^{{\rm{fcst}},{\rm{train}}}=F-{\langle {F}_{{\rm{train}}}\rangle }_{m,\tau }$.

### Fair-sliding

In one variant of *fair* tested here (*fair-sliding*), we maintain the same number of years in the reference climatology as *fair*, but the reference climatology is allowed to slide along behind each forecast (represented by dashed blue lines in Fig. Box 2), and thus does extend into the testing period for some forecasts (but still uses only observations available at the time the forecast is started). The reference climatology for *fair-sliding* always contains 17 years up to the year of the current forecast, *y*, such that the *fair-sliding* anomaly forecasts are ${f}^{{\rm{fcst}},y-16\to y}=F-{\langle {F}_{y-16\to y}\rangle }_{m,\tau }$.

### Fair-all

In another fair variant, (*fair*-*all*), the reference climatology includes all years in the training and testing period that precede the forecast, and thus the number of years in the reference climatology grows as the forecasts step through the testing period (Fig. Box 2, dashed orange lines). The first year of the training period is 1982, so the reference climatology for *fair*-*all* extends from then to the year of the current forecast, *y*. The *fair*-*all* anomaly forecasts are given by ${f}^{{\rm{fcst}},1982\to y}=F-{\langle {F}_{1982\to y}\rangle }_{m,\tau }$. A comparison of the performance of the *fair* variants relative to one another is given in Supplementary Note 4. The forecast anomalies generated for each of the *fair* variants are shown in Supplementary Note 5.

### Consistency of reference periods

When the forecasts are verified against observations, the observed anomalies use the same periods for the reference climatology as the forecasts. For example, for the *unfair* forecasts the observed anomalies are ${o}^{{\rm{obs}},{\rm{test}}}=O-{\langle {O}_{{\rm{test}}}\rangle }_{m}$. For the *fair* forecasts the observed anomalies are ${o}^{{\rm{obs}},{\rm{train}}}=O-{\langle {O}_{{\rm{train}}}\rangle }_{m}$. Similarly, the regression model forecasts are trained using consistent observed anomalies and periods as used by the model forecasts to which they are compared.

### Onset definition

**Fig. 6:**
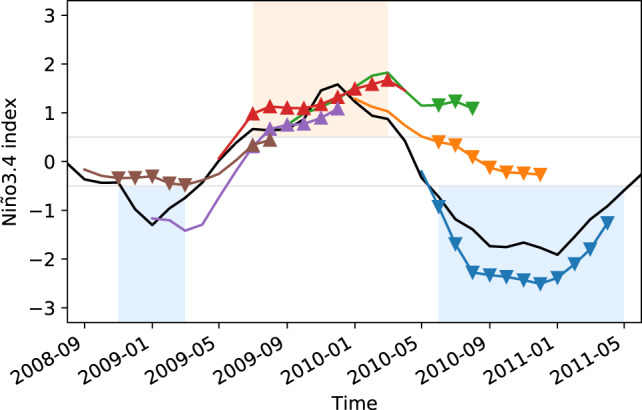
Illustration of El Niño and La Niña onset forecasts. For the 2010/11 El Niño/La Niña events we show some example forecasts from the cm4i model. The El Niño and La Niña events are defined from observations and denoted by red and blue shading respectively. To qualify as an El Niño/La Niña onset forecast, the forecast must have been initiated before the event occurred in observations (prior to and outside the red/blue area) and contain lead times that land during the period of the observed event (red/blue area). The model forecasts that land in the El Niño event time window are denoted by upward triangles and constitute the set of forecasts used to assess onset for the El Niño event shown. These may or may not correspond to El Niño events in the models. The set of forecasts that qualify for La Niña onset here are denoted by downward triangles.

Forecasting onset of ENSO events involves issuing a forecast of the event before the event has begun. We represent onset forecasts schematically for El Niño in Fig. 6. An onset forecast is any forecast that was initiated prior to an event and terminates during the event (where the event is defined in observations). We consider here only onset where the event occurs in observations and may or may not occur in models (true positives/false negatives). We do not examine forecasts of onset where the event occurs in models but not observations (false positives), for which the models may perform differently again.

## Supplementary information

Supplementary Information File

Editorial policy checklist file

## Data Availability

All data used here is available to the public. NMME forecasts are available from https://www.cpc.ncep.noaa.gov/products/NMME/. C3S forecasts are available from https://iridl.ldeo.columbia.edu/SOURCES/.EU/.Copernicus/.CDS/.C3S/. The Optimum Interpolation Sea Surface Temperature (OISST) data were downloaded from https://www.esrl.noaa.gov/psd/data/gridded/data.noaa.oisst.v2.html.

## Code availability

The analysis in this paper was carried out using the following open-source Python packages: doppyo (https://github.com/csiro-dcfp/doppyo), xskillscore (https://xskillscore.readthedocs.io/en/stable/), xarray (http://xarray.pydata.org/en/stable/), numpy (https://numpy.org/), dask (https://dask.org/), scipy (https://www.scipy.org/), scikit-learn (https://scikit-learn.org/), and matplotlib (https://matplotlib.org/).

## References

[1] Toth, Z. & Buizza, R. In *Sub-Seasonal to Seasonal Prediction: The Gap Between Weather and Climate Forecasting*, chap. 2, (eds Robertson, A. & Vitart, F.) 17–45 (Elsevier, New York, 2019). 585pp.

[2] Monselesan D, O’Kane T, Risbey J, Church J (2015). Internal climate memory in observations and models. Geophys. Res. Lett..

[3] Trenberth K (1997). The definition of El Niño. Bull. Amer. Met. Soc..

[4] Peña M, Kalnay E (2004). Separating fast and slow modes in coupled chaotic systems. Nonlinear Proc. Geoph..

[5] Yang S-C, Keppenne C, Rienecker M, Kalnay E (2009). Application of coupled bred vectors to seasonal-to-interannual forecasting and ocean data assimilation. J. Clim..

[6] Glantz, M. et al. *Teleconnections Linking Worldwide Climate Anomalies*, Vol. 535 (Cambridge University Press Cambridge, 1991).

[7] Wallace JM, Gutzler DS (1981). Teleconnections in the geopotential height field during the Northern Hemisphere winter. Mon. Weather Rev..

[8] Cane M, Zebiak S, Dolan S (1986). Experimental forecasts of El Niño. Nature.

[9] Barnston A (2010). Verification of the first 11 years of IRI’s seasonal climate forecasts. J. Appl. Meteorol. Climatol..

[10] Barnston AG, Tippett M, L’Heureux M, Li S, DeWitt D (2012). Skill of real-time seasonal ENSO model predictions during 2002–2011: is our capability increasing?. Bull. Amer. Met. Soc..

[11] Weisheimer A, Palmer T (2003). On the reliability of seasonal climate forecasts. J. R. Soc. Interface.

[12] Timmermann A (2018). El Niño–southern oscillation complexity. Nature.

[13] Dijkstra H, Petersik P, Hernandez-Garcia E, Lopez C (2019). The application of machine learning techniques to improve El Niño prediction skill. Front. Phys..

[14] Smith L, Du H, Suckling E, Niehörster F (2015). Probabilistic skill in ensemble seasonal forecasts. Q. J. R. Meteor. Soc..

[15] DelSole T, Tippett MK (2016). Forecast comparison based on random walks. Mon. Weather Rev..

[16] Kirtman B (2014). The North American multimodel ensemble: phase-1 seasonal-to-interannual prediction; phase-2 toward developing intraseasonal prediction. Bull. Amer. Met. Soc..

[17] L’Heureux M (2017). Observing and predicting the 2015/16 El Niño. Bull. Am. Meteorol. Soc..

[18] Glantz M (2015). Shades of chaos: lessons learned about lessons learned about forecasting El Niño and its impacts. Int. J. Disaster Risk Sci..

[19] Barnston AG, van den Dool H (1993). A degeneracy in cross-validated skill in regression-based forecasts. J. Clim..

[20] Lorenz, E. *The Essence of Chaos* (University of Washington Press, Seattle, 2004). 240pp.

[21] Goddard L (2013). A verification framework for interannual-to-decadal predictions experiments. Clim. Dynam..

[22] DelSole T, Shukla J (2009). Artificial skill due to predictor screening. J. Clim..

[23] McCullagh P (1980). Regression models for ordinal data. J. R. Stat.Soc..

[24] Mantua N, Hare S (2002). The pacific decadal oscillation. J. Oceanogr..

[25] Risbey J, Lewandowsky S, Hunter J, Monselesan D (2015). Betting strategies on fluctuations in the transient response of greenhouse warming. Phil. Trans. R. Soc. A.

[26] Stockdale T (1997). Coupled ocean-atmosphere forecasts in the presence of climate drift. Mon. Weather Rev..

[27] International Clivar Project Office. *Data and Bias Correction for Decadal Climate Predictions. Tech. Rep., World Climate Research Program*. (Clivar Publication Series No. 150, 2011).

[28] Boer G (2016). The decadal climate prediction project (DCPP) contribution to CMIP6. Geosci. Model Dev..

[29] Barnston AG, Tippett M, van den Dool H, Unger D (2015). Toward an improved multimodel ENSO prediction. J. Appl. Meteorol. Clim..

[30] Barnston AG, Tippett M, Ranganathan M, L’Heureux M (2019). Deterministic skill of ENSO predictions from the North American multimodel ensemble. Clim. Dynam..

[31] Parton K, Crean J, Hayman P (2019). The value of seasonal climate forecasts for Australian agriculture. Agricult. Sys..

[32] Fučkar N, Volpi D, Guemas V, Doblas-Reyes F (2014). A posteriori adjustment of nearterm climate predictions: Accounting for the drift dependence on the initial conditions. Geophys. Res. Lett..

[33] Choudhury D, Sen Gupta A, Sharma A, Mehrotra R, Sivakumar B (2017). An assessment of drift correction alternatives for CMIP5 decadal predictions. J. Geophys. Res..

[34] Kharin V, Boer G, Merryfield W, Scinocca J, Lee W (2012). Statistical adjustment of decadal predictions in a changing climate. Geophys. Res. Lett..

[35] Cannon A (2018). Multivariate quantile mapping bias correction: an N-dimensional probability density function transform for climate model simulations of multiple variables. Clim. Dynam..

[36] Banzon V, Smith T, Chin T, Liu C, Hankins W (2016). A long-term record of blended satellite and in situ sea-surface temperature for climate monitoring, modeling and environmental studies. Earth System Science Data.

[37] Rayner N (2003). Global analyses of sea surface temperature, sea ice, and night marine air temperature since the late nineteenth century. J. Geophys. Res..

[38] Manzanas R (2019). Bias adjustment and ensemble recalibration methods for seasonal forecasting: a comprehensive intercomparison using the C3S dataset. Clim. Dynam..

[39] Kumar A, Hu Z, Jha B, Peng P (2017). Estimating ENSO predictability based on multi-model hindcasts. Clim. Dynam..

[40] Lepore C, Tippett M, Allen J (2017). ENSO-based probabilistic forecasts of March–May U.S. tornado and hail activity. Geophys. Res. Lett..

[41] Tippett M, Ranganathan M, L’Heureux M, Barnston A, DelSole T (2019). Assessing probabilistic predictions of ENSO phase and intensity from the North American multimodel ensemble. Clim. Dynam..

[42] DelSole T, Tippett MK (2014). Comparing forecast skill. Mon. Weather Rev..

[43] Jolliffe I, Stephenson D (2012). Forecast verification: a practioner’s guide in atmospheric science.

[44] Kirtman B (2003). The COLA anomaly coupled model: Ensemble ENSO prediction. Mon. Weather Rev..

[45] Saha S (2006). The NCEP climate forecast system. J. Clim..

[46] Kirtman B, Min D (2009). Multimodel ensemble ENSO prediction with CCSM and CFS. Mon. Weather Rev..

[47] Smith D (2007). Improved surface temperature prediction for the coming decade from a global climate model. Science.

[48] Hudson D, Alves O, Hendon H, Marshall A (2011). Bridging the gap between weather and seasonal forecasting: intraseasonal forecasting for Australia. Q. J. R. Meteor. Soc..

[49] Hudson D, Marshall A, Alves O (2011). Intraseasonal forecasting of the 2009 summer and winter Australian heat waves using POAMA. Weather Forecast..

[50] Cottrill A (2013). Seasonal forecasting in the Pacific using the coupled model POAMA-2. Weather Forecast..

[51] Hudson D, Marshall A, Yin Y, Alves O, Hendon H (2013). Improving intraseasonal prediction with a new ensemble generation strategy. Mon. Weather Rev..

[52] Xue Y, Chen M, Kumar A, Hu Z-Z, Wang W (2013). Prediction skill and bias of tropical Pacific sea surface temperatures in the NCEP climate forecast system version 2. J. Clim..

[53] Marshall A (2014). Intra-seasonal drivers of extreme heat over Australia in observations and POAMA-2. Clim. Dynam..

[54] White C, Hudson D, Alves O (2014). ENSO, the IOD and the intraseasonal prediction of heat extremes across Australia using POAMA-2. Clim. Dynam..

[55] Zhou Y, Kim H (2018). Prediction of atmospheric rivers over the North Pacific and its connection to ENSO in the North American multi-model ensemble (NMME). Clim. Dynam..

[56] O’Kane T (2019). Coupled data assimilation and ensemble initialization with application to multiyear ENSO prediction. J. Clim..

[57] Maraun D, Widmann M (2018). Cross-validation of bias-corrected climate simulations is misleading. Hydrol. Earth Syst. Sci..

[58] Wang W, Chen M, Kumar A (2010). An assessment of the CFS real-time seasonal forecasts. Weather Forecast..

[59] Tippett M, Barnston A, Li S (2012). Performance of recent multimodel ENSO forecasts. J. Appl. Meteorol. Clim..

