# Supplementary material for: Standard assessments of climate forecast skill can be misleading
Source: Nat Commun. 2021 Jul 16;12:4346. doi: 10.1038/s41467-021-23771-z (PMC8285537; doi:10.1038/s41467-021-23771-z)
Supplement: Supplementary file 1 — Supplementary Information File [file 41467_2021_23771_MOESM1_ESM.pdf]

Supplementary material

Standard assessments of climate forecast skill can be misleading

James S. Risbey[1,*,†], Dougal T. Squire[1,†], Amanda S. Black[1], Timothy DelSole[4], Chiara Lepore[5], Richard J. Matear[1], Didier P. Monselesan[1], Thomas S. Moore[1], Doug Richardson[1], Andrew Schepen[2], Michael K. Tippett[3], and Carly R. Tozer[1]

[1]CSIRO Oceans & Atmosphere, Box 1538 Hobart, Tas 7001, Australia
[2]CSIRO Land & Water, Brisbane, Qld, Australia
[3]Department of Applied Physics and Applied Mathematics, Columbia University, New York, NY, USA
[4]Department of Atmospheric, Oceanic, and Earth Sciences, George Mason University, Fairfax, VA, USA
[5]Lamont-Doherty Earth Observatory, Columbia University, Palisades, NY, USA
[*]Corresponding author: ✉ james.risbey@csiro.au
[†]Co-first author

1 Supplementary Note 1 : Model skill measures

A skill score for a forecast quantifies how well the forecast did in repeated trials relative to a well defined reference forecast. There are many different measures of model skill. Different measures reward and penalise different attributes of a forecast, and thus a particular model may do well on some measures but not others [24]. It is important therefore to use a range of skill measures. In the main text we used the random walk skill score, which is well suited for the comparison of forecast systems. It is also desirable to provide a measure of skill relative to simple baselines such as chance or past averages.

One such measure is the Gerrity score (GS) [60], which assesses ENSO as a categorical forecast (El Niño, neutral, La Niña) based on which range the Niño3.4 value lies. Another measure is the mean squared skill score ($MSSS$) [61], which assesses the forecast according to how far the forecast Niño3.4 values lie from the observed values, and is thus referred to as a distance-based skill score. We assess the $MSSS$ for each model forecast relative to a climatological forecast (where likelihoods are set relative to past behaviour). The GS and $MSSS$ skill scores for ENSO for a set of dynamical models and for a linear and logistic regression model are shown in Supplementary Figure 1.

For categorical forecasts, all models are better than chance for leads out to about 10 months. The dynamical models have mostly similar categorical scores to the linear and logistic regression models (given allowance for sampling uncertainty). For the distance-based skill measure, all models are better than a climatological forecast for about 6 months. Subsets of the dynamical models are partly similar, and partly worse than the linear regression model. The cola model is an outlier with less skill at short lead times (< 3 months) than all other models for both categorical and distance-based skill scores. This is related to a discontinuity in initial conditions for this model (see Supplementary Note 2).

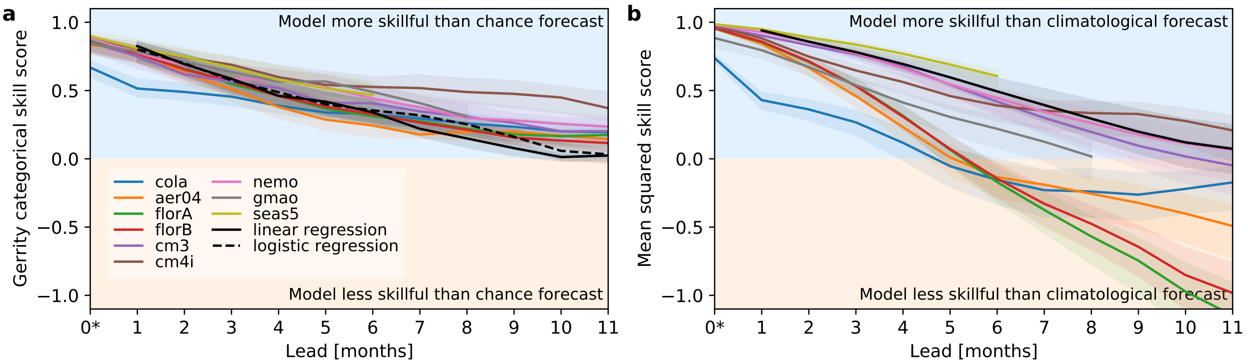

Supplementary Figure 1: Categorical and distance-based skill scores. Panel a shows the Gerrity score (GS), which assesses whether the forecast is better than chance in predicting the correct category for Niño3.4. For a three category classification of ENSO (El Niño, neutral, La Niña) this score is defined as $GS = \frac{1}{n}\sum_{i=1}^{3}\sum_{j=1}^{3} q(f_i, o_j)s_{ij}$, where $q(f_i, o_j)$ is the number of forecasts in category i that had observations in category j and s_{ij} are elements of a scoring matrix. The scoring matrix tabulates the reward or penalty for each of the nine possible combinations in forecasting one of 3 categories and observing one of 3. s_{ij} is configured such that $GS = 0$ for random and constant forecasts. The chance forecast provides an equal weighting to each of the three El Niño Southern Oscillation (ENSO) categories. For GS, a perfect forecast scores 1 and a chance forecast scores 0. Panel b shows the mean squared skill score ($MSSS$), which measures the mean squared difference between the Niño 3.4 forecasts and observations relative to a forecast of the climatological value of Niño 3.4 for each month. The $MSSS$ is based on the mean square error (MSE). For a set of instances, i, of n matched forecasts, f_i, and observations, o_i, $MSE(f, o) = \frac{1}{n}\sum_{i=1}^{n}(f_i - o_i)^2$. The $MSSS$ can then be standardised as the difference between the forecast in question and a reference forecast, r, normalised by the difference between a perfect forecast, p, and the reference forecast: $MSSS(f, r, p, o) = [MSE(f, o) - MSE(r, o)]/[MSE(p, o) - MSE(r, o)]$. Since $MSE(p, o) = 0$ for a perfect forecast, then $MSSS(f, r, o) = 1 - [MSE(f, o)/MSE(r, o)]$, where r here is taken to be a climatological forecast for each month, m. For $MSSS$ a perfect forecast scores 1 and a climatological forecast scores 0. The dynamical models are represented by the colored lines in the legend and the statistical models are the black lines. The shaded bands on each line represent a 5–95% range of values from a bootstrap resampling. Sampling variability is approximated from 1000 resampled estimates of the skill metric, generated by bootstrapping across forecast and ensemble member (prior to computing the ensemble mean). The skill scores are calculated using *fair* anomalies for hindcasts over the test period 1999–2015.

2 Supplementary Note 2 : Initial condition discontinuity

The cola model has a discontinuity in the supplied initial conditions for its forecasts around 1999 [15, 37, 39]. This discontinuity is potentially important as the reference period used in this work is prior to 1999 and the testing period is after 1999. This discontinuity applies only to the cola model and will degrade skill of the cola model. We keep the cola model in the set of models here as it illustrates the role of initial condition errors relative to biases in forecast climatology with lead time. The discontinuity is evident by plotting the difference (error) between each model's initial Niño3.4 value and the observed value. This plot is shown in Supplementary Figure 2. For a two-sample Kolmogorov-Smirnov test on the 1982–1998 and 1999–2015 error distributions, for cola and gmao the null hypothesis (that the distributions are identical) is rejected at a p-value of 0.01. The error distributions of each period are indistinguishable at this p-value for all other models. For the other models, any change in model bias between the training and testing periods is not due to changes in initial condition error.

The cola model has some (non-zero) change in model bias, ΔB, at low lead (leads 0^*, 1), whereas the other models have almost no change in bias at this range. This can be seen clearly in row g of Fig. 4 for the month of March for example, where most models (except cola) have almost no change in bias until lead 3. The issue is not simply that cola has some bias at low lead, as that is also true of the aer04 model for example. The bias at low lead is consistent from training to testing period in the aer04 model so that its change in bias is small. For cola the bias is not consistent at low lead from training to testing period, resulting in a larger change in bias at low lead. The change in bias at low lead explains why cola performs poorly using *fair*-based methods at low lead times relative to the other models.

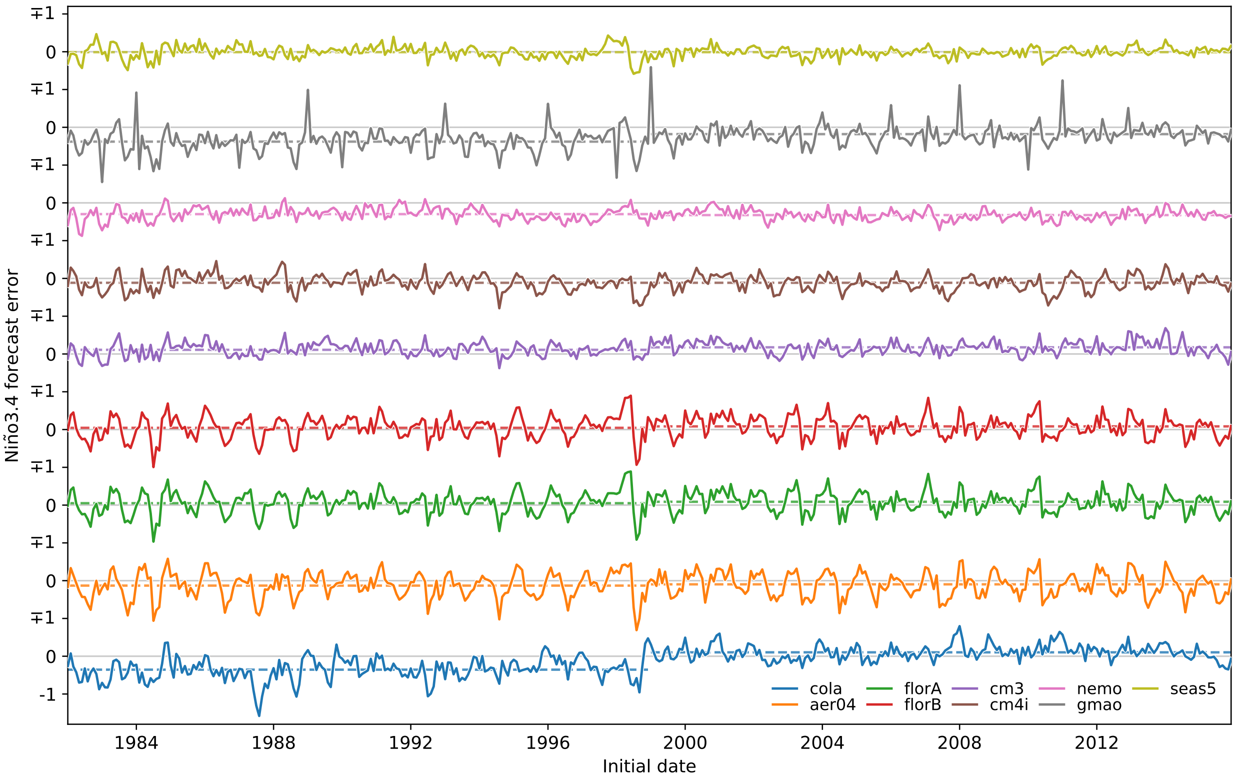

Supplementary Figure 2: Forecast error (forecast Niño3.4 − observed Niño3.4) at lead 0^* for set of models examined. The solid line is the error for every month. The dashed line is the mean error for the time periods 1982–1998 and for 1989–2016.

3 Supplementary Note 3 : Comparison of dynamical model with logistic regression model

One might argue that the use of a distance measure to score forecasts in Fig. 2 favours the linear regression model, which is based on a fit that minimizes the distance of the regression fit with the observations. The dynamical models are of course not explicitly designed to 'fit' ENSO in this way. To provide an alternative regression that is not based on a distance fit, we show the random walk skill scores for a comparison with the categorical logistic regression model in Supplementary Figure 3 panels a–e. In this case all ENSO forecast anomalies are converted to ENSO categories, and the skill score rewards winning forecasts in the correct category. The comparison of the linear regression forecasts with the logistic regression forecasts is represented by the black line, which is squarely in the white 'chance' zone indicating that neither forecast is superior. For the *fair* variants, the dynamical models are either in the white zone or on the boundary between this zone and the zone where the models are less skillful than the logistic regression. This is a marginally better result for the dynamical models than for the comparison with linear regression (Fig. 2), but does not change the qualitative conclusion that the dynamical models are not outperforming the simple regression forecasts. The results for lead 3 months are repeated for all lead times between 1 and 11 months in panels f–j. The random walk skill score is mostly constant as a function of lead time for the categorical forecast comparisons.

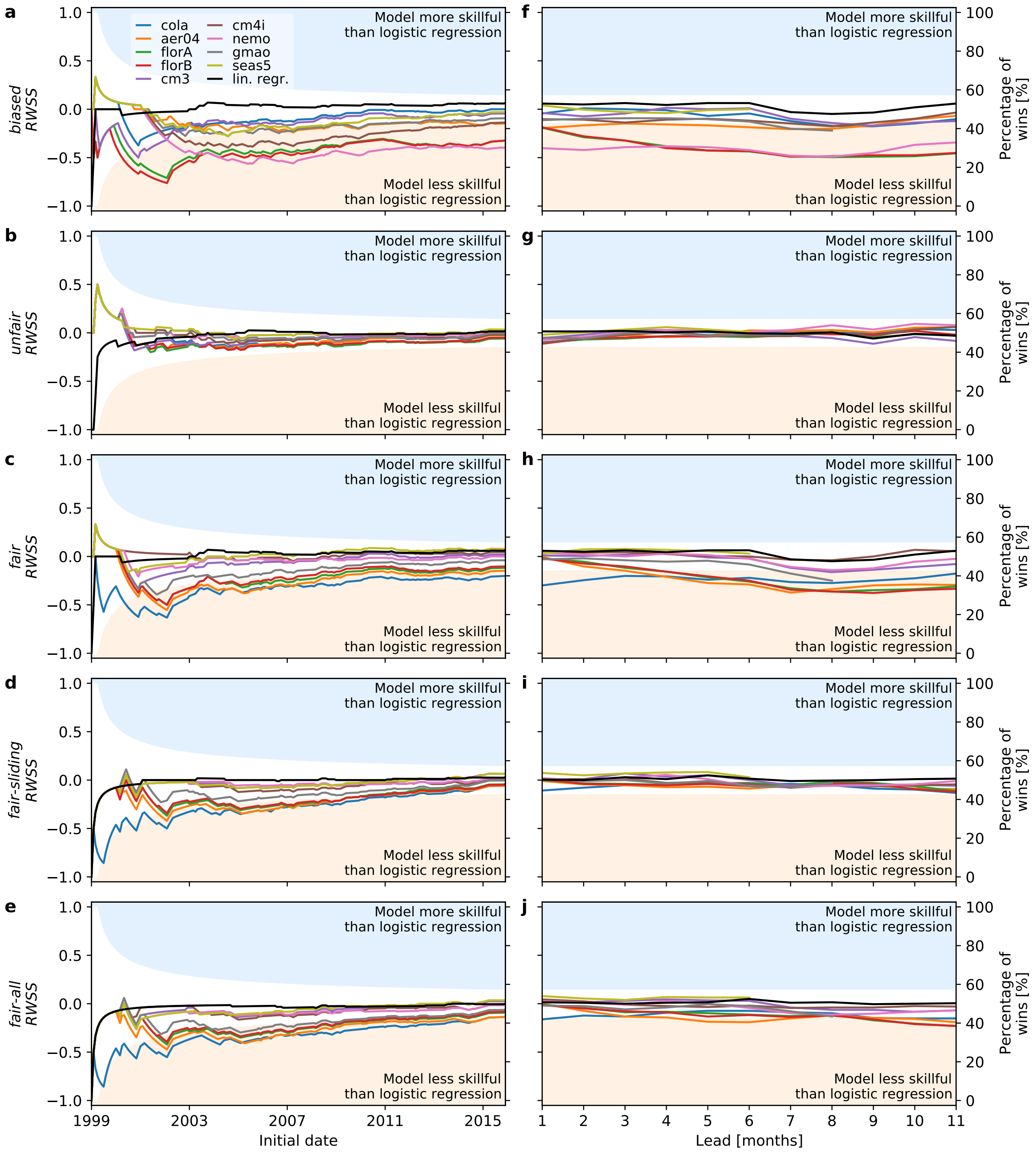

Supplementary Figure 3: Skill relative to logistic regression. This plot is equivalent to Fig. 2, but for logistic regression instead of linear regression. Panels a–e show dynamical model Niño3.4 random walk skill score ($RWSS$) at lead 3 months relative to logistic regression model using the methods: *biased*, *unfair*, *fair*, *fair-sliding*, and *fair-all*. Forecasts are for the period 1999 to 2016. The panels f–j show the value of the $RWSS_n$ for lead times from 1–11 months. The black line represents the score for the linear regression model relative to the logistic regression model. The coloured lines refer to dynamical models as in the legend.

4 Supplementary Note 4 : Comparison of fair variants

In this section we compare three simple fair variants (*fair*, *fair-sliding*, *fair-all*) using the same forecast systems. The comparison of *fair* and *fair-sliding* is shown in panels a, b, and c of Figure Supplementary 4. For the more sensitive models displaying larger change in bias from training to testing periods (aer04, cola, florA, florB), at lead 3 and lead 6 (panels a and b), the random walk score tends to progressively favour *fair-sliding* over *fair* as more and more forecast comparisons are made later and later in the hindcast period. Each successive forecast comparison steps further away from the period used to define the reference climatology for *fair*, whereas *fair-sliding* moves the reference climatology along behind the forecast and so continues to do better and better relative to *fair*. The random walk scores as a function of lead time are shown in panel c. For almost all the models there is progressively more advantage to *fair-sliding* relative to *fair* as lead time increases. Keeping the reference climatology current (*fair-sliding*) is increasingly important at longer leads.

The results for comparison of *fair-sliding* and *fair-all* are shown in panels d, e, and f. The more sensitive models (aer04, florA, florB) get progressively better for *fair-sliding* relative to *fair-all* with successive forecast comparisons. That is to say, these models receive more of an advantage by keeping their reference climatology current (as close as possible to the forecast time) than by growing a larger and larger reference climatology even though it extends to the time of the forecast. The extra information in the longer *fair-all* reference climatology (that is further away from the forecast time) is actually degrading the skill in these models. The skill advantage for *fair-sliding* over *fair-all* is mostly manifest at longer lead times. For the least sensitive models (cm3, cm4i), skill tends to be higher when the longer reference climatology (*fair-all*) is used.

In summary, the 'fair' method yielding highest skill for the dynamical model hindcasts is *fair-sliding*, where the reference climatology slides along behind the forecast being made. This method is generally superior to even one in which all prior forecasts are used in forming the reference climatology. This result can be partly understood in terms of the periods tested. Most of the model forecast climatologies exhibit warming from the training to testing period, whereas the observations cool slightly over these periods. These differences between observed and model climatological trends will contribute to large bias errors for a static reference climatology like *fair* [53]. The result favouring *fair-sliding* is probably also a particular feature of ENSO hindcasts/forecasts because of the nature of ENSO. That is, ENSO hindcasts are particularly challenging to evaluate for skill because the available time series to develop the model hindcast climatology is somewhat pathological in containing small numbers of very large events. As one changes forecast periods from one decade to another, one can easily pick up a large new event (big El Niño or La Niña) in a forecast testing period that is not well sampled from the forecast climatology over prior periods. That makes it challenging to develop a reference climatology and increases the sensitivity of bias-corrected skill to choice of reference period. As such, it pays for the reference climatology to be as close as possible to the testing period. For real forecasts, one might effectively decrease the contribution of bias errors to forecast degradation by updating the reference climatology to be as current as possible every time a new forecast is launched.

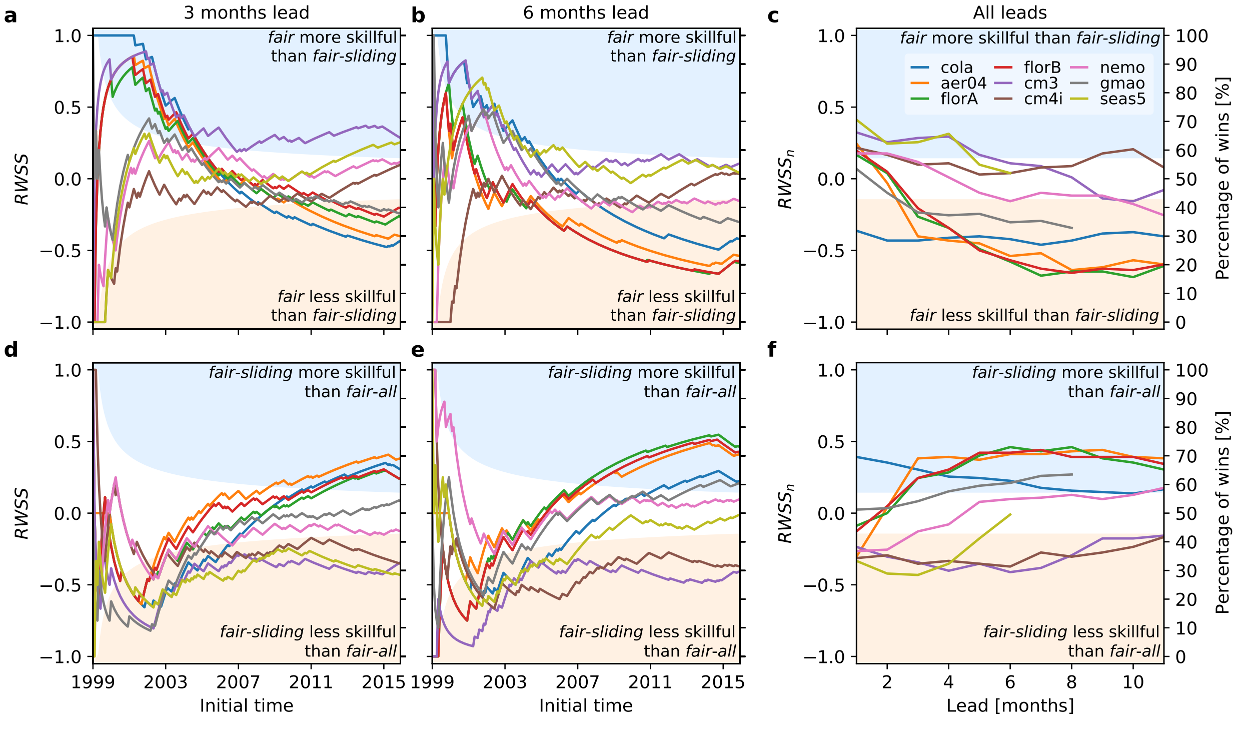

Supplementary Figure 4: Comparison of El Niño Southern Oscillation (ENSO) skill for *fair* and *fair-sliding* anomaly calculation (panels a, b, c), and for *fair-sliding* and *fair-all* (panels d, e, f). The plots show random walk skill scores ($RWSS$) for lead 3 (panels a and d), lead 6 (panels b and e), and for final random walk scores ($RWSS_n$) as a function of lead time (panels c and f).

5 Supplementary Note 5 : Forecast anomalies

The mean of the forecast anomalies as a function of lead time and calendar month, $\langle f^{fcst} \rangle_{m,\tau}$, is shown in Supplementary Figure 5 for each of the 'fair' methods here (*fair*, *fair-all*, *fair-sliding*). For 4 of the 9 models (aer04, cola, florA, florB) the mean anomalies are biased large and positive, which degrades their forecast skill. The warm biases in the mean anomalies are largest for the *fair* method (row a) and smallest for *fair-sliding* (row c), consistent with the relative skill between the 'fair' methods shown in Supplementary Figure 4. The cm3, cm4i, nemo, and seas5 models are less sensitive than the other models in that they are least affected by the choice of reference period. This is related to the degree of constancy of climatological bias in these models.

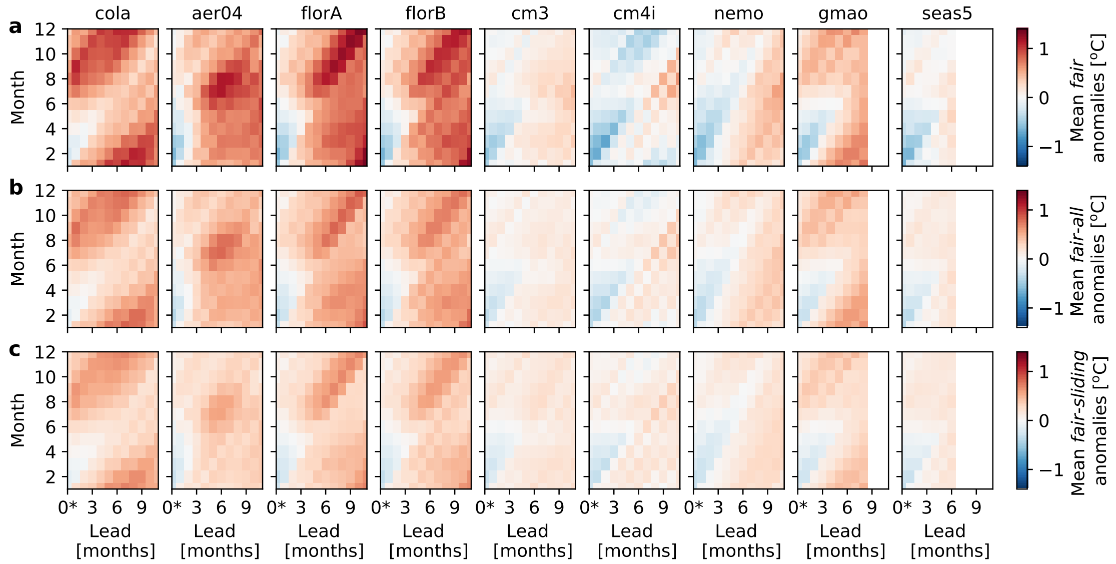

Supplementary Figure 5: Mean Niño3.4 forecast anomalies as functions of lead time and calendar month for (a) *fair*, (b) *fair-all* and (c) *fair-sliding* methods.

6 Supplementary Note 6 : Fair versus cross-validated unfair method

The figure below provides a comparison of *fair* and cross-validated unfair, *unfair-cv*, hindcasts. The results are substantially the same as those for the comparison of *fair* and straight *unfair* in Fig. 3, indicating that the application of cross-validation does little to make *unfair* hindcasts fairer.

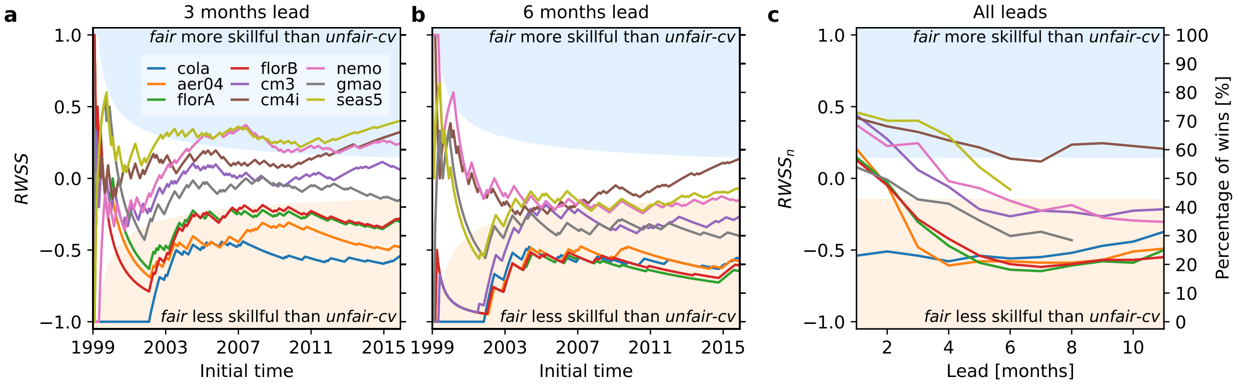

Supplementary Figure 6: Comparison of ENSO skill for *fair* and *unfair-cv* cross-validated methods. $RWSS$ for lead 3 (a), lead 6 (b), and $RWSS_n$ as a function of lead time (c). This plot is equivalent to Fig. 3, except that cross-validation is applied to the *unfair* method, where a centred period of 7 years have been removed in deriving the reference climatology for each year.

7 Supplementary Note 7 : Skill of onset forecasts for unfair method

Supplementary Figure 7 shows the skill of dynamical model forecasts of onset of El Niño and La Niña relative to linear regression forecasts using the *unfair* method. For this method the results don't change much from the original periods (panels b and e) to the switched periods (panels c and f). This stands in contrast to results using the *fair* method (Fig. 5), where the lead-time dependent changes in bias across different periods can act to help or hinder a forecast of onset.

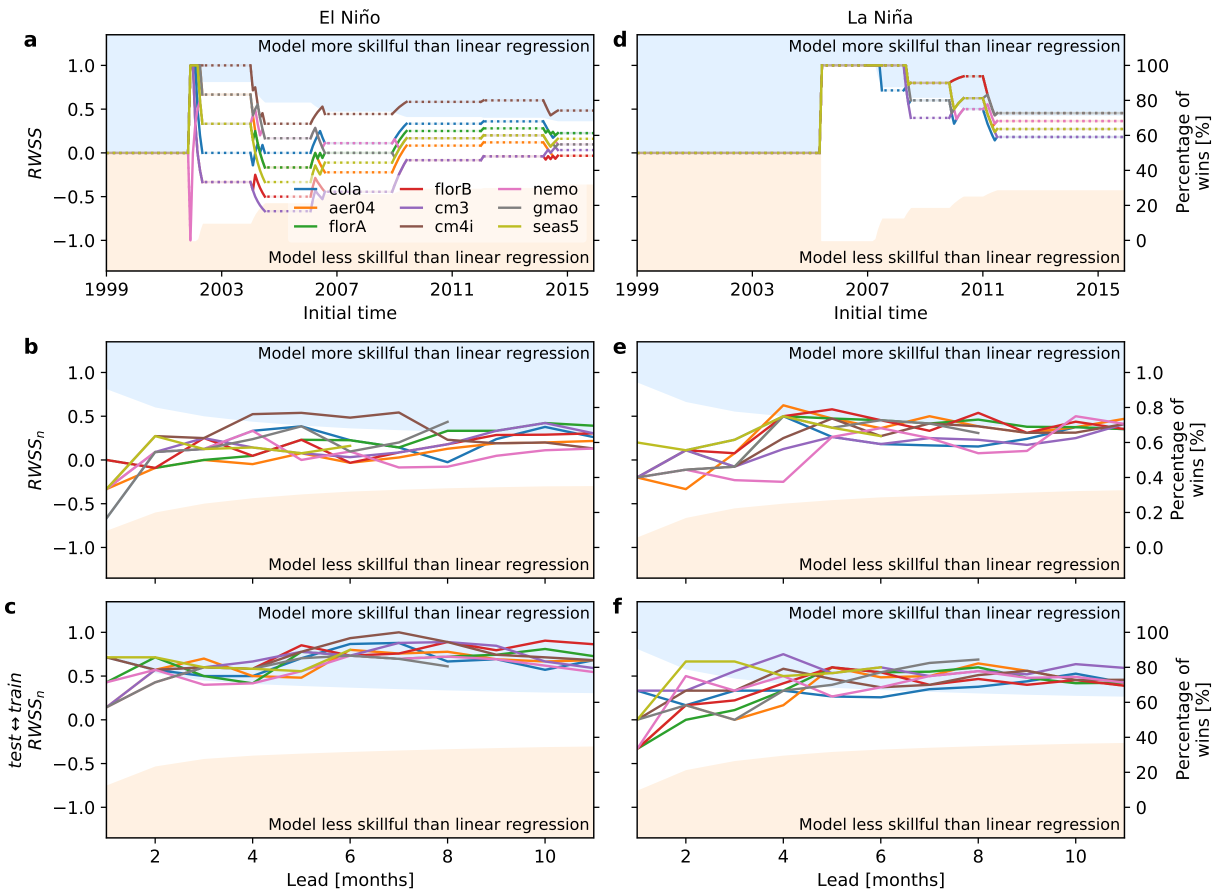

Supplementary Figure 7: Skill scores for El Niño and La Niña onset using the *unfair* method. Random walk skill score ($RWSS$) at 6 months lead relative to linear regression for El Niño and La Niña (a and d). The dashed lines in the panel represent forecast comparison periods that do not correspond to 'onset' comparisons. $RWSS_n$ as a function of lead time for El Niño and La Niña (b and e). Panels c and f are the same as panels b and e, except that the training and testing periods have been switched.
